# Species, Sequence Types and Alleles: Dissecting Genetic Variation in *Acanthamoeba*

**DOI:** 10.3390/pathogens9070534

**Published:** 2020-07-02

**Authors:** Paul A. Fuerst, Gregory C. Booton

**Affiliations:** 1 Department of Evolution, Ecology and Organismal Biology, The Ohio State University, Columbus, OH 43210, USA; 2 Department of Molecular Genetics, The Ohio State University, Columbus, OH 43210, USA; Booton.1@osu.edu

**Keywords:** *Acanthamoeba*, free-living amoebae, sequence types, species, alleles, nuclear 18S rRNA gene, DNA sequences, ASA.S1

## Abstract

Species designations within *Acanthamoeba* are problematic because of pleomorphic morphology. Molecular approaches, including DNA sequencing, hinted at a resolution that has yet to be fully achieved. Alternative approaches were required. In 1996, the Byers/Fuerst lab introduced the concept of sequence types. Differences between isolates of *Acanthamoeba* could be quantitatively assessed by comparing sequences of the nuclear 18S rRNA gene, ultimately producing 22 sequence types, designated T1 through T22. The concept of sequence types helps our understanding of *Acanthamoeba* evolution. Nevertheless, substantial variation in the 18S rRNA gene differentiates many isolates within each sequence type. Because the majority of isolates with sequences in the international DNA databases have been studied for only a small segment of the gene, designated ASA.S1, genetic variation within this hypervariable region of the 18S rRNA gene has been scrutinized. In 2002, we first categorized variation in this region in a sample of T3 and T4 isolates from Hong Kong, observing ten “alleles” within type T4 and five “alleles” within T3. Subsequently, confusion occurred when different labs applied redundant numerical labels to identify different alleles. A more unified approach was required. We have tabulated alleles occurring in the sequences submitted to the international DNA databases, and determined their frequencies. Over 150 alleles have occurred more than once within 3500+ isolates of sequence type T4. Results from smaller samples of other sequence types (T3, T5, T11 and T15, and supergroup T2/6) have also been obtained. Our results provide new insights into the evolutionary history of *Acanthamoeba*, further illuminating the degree of genetic separation between significant taxonomic units within the genus, perhaps eventually elucidating what constitutes a species of *Acanthamoeba*.

## 1. Introduction

The classification of *Acanthamoeba* as a distinct genus can be traced to a series of four notes by Aldo Castellani related to an amoeba contaminating a culture of *Cryptococcus pararoseus* [1,2,3,4]. The amoeba was subsequently described and classified as a new form, “*Hartmannella castellanii*” [5]. The amoeba that Castellani had observed was then identified by Volkonsky as distinct from other amoebae classified within the genus *Hartmannella*, leading to a split in the genus with the proposal of a new genus, *Acanthamoeba* [6]. Until the mid-1970s, the classification of amoebae to *Acanthamoeba* was dependent upon morphological criteria such as mitotic pattern, including spindle shape [6,7], physical structure of either the trophozoite or cyst stages [8,9,10,11], or some attempts to define amoebic cellular cytochemistry [8,11]. Subsequently, methods including immunochemistry [12] and protein electrophoresis [13] began to be employed to better understand the relationships between the amoebae classified within *Acanthamoeba*.

The use of DNA-based methods commenced during the 1970s, starting with DNA–DNA hybridization [14]. Attempts to classify and relate isolates of *Acanthamoeba* in the early 1980s made use of advances in molecular biology, centering on the development of DNA-based methods of phylogenetic analysis such as RFLP analysis of mitochondrial DNA [15]. By the end of the 1980s, DNA sequencing technology had advanced to the point where researchers could conceive of collecting extensive genetic information to inform us about the relationships between isolates, hopefully leading to a better understanding of what constitutes a “species” of *Acanthamoeba*. The methods further offered the possibility that we could begin to unravel the levels of genetic variability that exist within *Acanthamoeba sensu lato*. Here, we will review how the understanding of genetic variation within *Acanthamoeba* progressed and how it has advanced in the era of DNA sequencing. 

## 2. Species of *Acanthamoeba*

### 2.1. Species Pre-1980

Morphological approaches, primarily those based on trophozoite and/or cyst morphology, resulted in the description of more than ten species of *Acanthamoeba* by the middle of the 1970s (Table 1). The generic classification of the five species described prior to 1960 was contentious, since some researchers argued that *Acanthamoeba* was not a valid genus, but was merely a subgroup within the genus *Hartmannella* [7,16]. Subsequent studies by Page [8,17] were critical, by convincingly showing that morphological criteria were sufficient to show that “species” of *Acanthamoeba* were distinct from members of *Hartmannella*.

The next major contribution to the classification of species was made by Pussard and Pons [9], who primarily used cyst morphology to classify taxa of *Acanthamoeba*. While reviewing samples of most of the forms of *Acanthamoeba* that had been characterized previously, they also defined eight new species. Although this study almost doubled the number of named species of *Acanthamoeba*, its greatest impact was accomplished by describing characteristics that subdivided the genus *Acanthamoeba* into three distinct groups, based primarily on trophozoite and cyst sizes and cyst morphology. For more extensive details on the differences that they defined to distinguish between groups, see the original Pussard and Pons paper. The new species identified by Pussard and Pons are listed in Table 2.

Group I amoebae have the largest cysts, and group I species can easily be recognized from the star aspect of the endocyst. Other characteristics of group I cysts also separate these cells from other groups. Pussard and Pons classified the pre-existing taxa *A. astronyxis* and *A. comandoni* into group I.

Group II includes the forms that are most commonly encountered in various studies of clinical or environmental isolates. The group included *A. castellanii*, *A. polyphaga*, *A. rhysodes* and *A. griffini,* and six species shown in Table 2. Cysts are of small or medium size. Endocysts are never so distinctly star like as in group I; they are more open globular, ovoid, or polyhedral.

Finally, group III included *A. palestinensis, A. culbertsoni, A. lenticulata,* and *A. royreba*, in addition to the single species (*A. pustulosa)* listed in Table 2. Cysts of group III acanthamoebae are of medium size or smaller, and have a globose or ovoid endocyst, never starred. The absence of star-shaped endocysts in culture provides an important feature that established a break between group III and group II. Pussard and Pons acknowledge the difficulty of absolutely separating groups II and III when they stated that in some of its characteristics, *A. palestinensis* is a transitional species between group II and group III.

One additional species was proposed in 1979, *A. tubiashi*, a group I form, with type isolate OC-15C (ATCC 30867) [28]. This represented the last species description that did not make reference to the use of molecular/genetic criteria to describe a “species” of *Acanthamoeba.*

### 2.2. Species 1980–2000

More than a decade would pass before the next additions to the list of proposed species of *Acanthamoeba.* The 1980s would mark the incorporation of protein and nucleic acid genetic markers into the phylogenetic study of species. For multicellular eukaryotes, this transition had started in the late 1960s, when non-denaturing protein electrophoresis had begun to be incorporated into the study of genetic variation in populations [29,30,31,32,33]. Subsequently, these methods were used in the study of species relationships [34,35,36]. Protein electrophoresis was implemented to study *Acanthamoeba* a decade later [13], and was utilized, in conjunction with morphological information, for the descriptions of four new proposed species during the 1990s (Table 3).

Although no new species were described during the 1980s, research on species/strain relationships between isolates of *Acanthamoeba* began to incorporate more powerful molecular tools, especially methods of analysis of DNA differences. Again, the application of new resources to the study of *Acanthamoeba* lagged behind studies on multicellular eukaryotes. These newer techniques included Restriction Fragment Length Polymorphism (RFLP) analysis of mitochondrial DNA [15,37], the use of RFLP analysis (ribotyping) of nuclear 18S rDNA sequences [38,39], and DNA sequencing of the 18S rRNA gene [40]. Ultimately, DNA sequencing of the 18S rRNA gene of *Acanthamoeba* isolates became the gold standard to characterize any strain that had been isolated from clinical or environmental sources.

### 2.3. Species Post-2000

In the period since 2000, the proposals of new species of *Acanthamoeba* have all included information on the sequence of the 18S rRNA gene of the type isolate as a major, or even THE major, data point to justify the proposal of a new species. Four new species have been proposed since 2000, and are listed in Table 4. Other information, such as trophozoite size and cyst morphology, is also incorporated in each species description. Nevertheless, it appears that, in 2020, the sentiment of most investigators is that sequence differentiation from all previous forms would be required for a new species to be proposed. However, the degree of sequence differentiation remains imprecise.

One additional “species” of *Acanthamoeba* should be mentioned. In 1979, a new member of the family Acanthamoebidae was described [49]. This amoeba was placed into a new genus, *Comandonia*, proposed to be closely related to *Acanthamoeba*. The species was designated *Comandonia operculata*. The morphological analysis of trophozoites and cysts suggested that this amoeba fell outside the usual boundaries of the genus *Acanthamoeba*. It does not appear that a type specimen for the original strain, *C. operculata* L5C, still exists in a culture collection or laboratory that can be directly related to the original isolate. However, another isolate exists which is assumed to fit within the morphological descriptions of the species, *C. operculata* CDC-149 (ATCC 50243). This isolate was studied using molecular methods by Amaral Zettler and colleagues, and their results were presented at the IXth International Conference on Free-Living Amoebaein Paris in 2001. Based on the sequence of the 18S rRNA gene, the isolate, *C. operculata* CDC-149, was placed solidly within the genetic bounds of the genus *Acanthamoeba* [50]. Because of these results, *Acanthamoeba* (formerly *Comandonia*) *operculata* is considered here to be an additional described species of *Acanthamoeba*.

As we enter the third decade of the twentieth century, 31 species have been described and placed within the genus *Acanthamoeba*. What does species designation within *Acanthamoeba* reflect? It has been known for many years that morphology of trophozoites for isolates of *Acanthamoeba* can be very flexible, and cyst morphology may also be subject to environmental and subtle genetic effects. Do “species” have very different pathogenic potential? Could associations that may be proposed between different nominal species and various disease conditions suggest alternative approaches to treatment of the diseases? Does a species name actually designate a valid evolutionary lineage? An approach using DNA sequences for the analysis of isolates of *Acanthamoeba* began to provide new insights to answer such questions.

## 3. Sequence Types of *Acanthamoeba*

### 3.1. The Ribosomal Small Subunit rRNA Gene as a Focus of Study

During the 1990s, as the use of the Polymerase Chain Reaction (PCR) [51,52] became widespread, there was an increasing consensus that DNA sequences from the ribosomal small subunit rRNA gene (*Rns*) could provide important, even decisive, information concerning the classification of microbial isolates. Collection of *Rns* sequences became the standard approach to bacterial systematics. The gene occurs universally in all eubacteria, archaea and eukaryotes. The nucleotide sequence varies in a manner that indicates that the gene is not unusually conservative in change, and that studies of differences will yield information about the evolutionary relationships between forms.

How extensive has the study of the small subunit rRNA gene been over the last 30+ years? A quick scan of the sequences deposited by the end of February 2020 into the international DNA databases (as listed by GenBank) suggests that more than 9 million *Rns* sequences (including eubacterial and archaeal 16S rRNA sequences and eukaryotic nuclear 18S rRNA and mitochondrial 16S-like rRNA sequences) have been deposited, a number that would actually underestimate the number of such sequences that have been determined by researchers over the last 30 years. A further search of GenBank indicates that approximately 9000 of these sequences come from studies of the 18S rRNA gene from organisms designated as belonging to the Amoebozoa. Finally, slightly more than 3600 of the Amoebozoan 18S rRNA sequences are attributed to isolates of *Acanthamoeba.* Researchers from the *Acanthamoeba* FLA community have been very busy. Note that our own database at The Ohio State University (OSU) indicates that the number of *Rns* gene sequences from *Acanthamoeba* actually exceeds 5000, since a substantial number of isolates remain undeposited in the international DNA databases. Our studies utilize the OSU database for analyses [53].

### 3.2. The Development of the Concept of a “Sequence Type” in Acanthamoeba

The initial DNA sequence of an 18S rRNA gene from any isolate of *Acanthamoeba* was reported for the Neff isolate of *A. castellanii* in 1986 [40]. This *Rns* sequence indicated that the gene in *Acanthamoeba* was unlike that found in most other eukaryotes, with a deposited sequence 2303 nucleotides in length. This made the *Acanthamoeba* gene unusually long, since most eukaryotic *Rns* sequence are between 1800 and 1900 bases in length; for example, the human 18S rRNA gene sequence is 1870 bases in length [54]. The extra sequences within the *Acanthamoeba* gene, referred to as expansion segments, are found dispersed throughout several parts of the molecule. Subsequent work indicated that they remain within the rRNA transcript, and are not excised, as would occur if they represented intron sequences. A proposed secondary structure was eventually presented, which shows the dispersal of the expansion segments [55]. The expansion segments would ultimately provide the justification for focusing on 18S rRNA gene sequences as an important focus in the study of isolates of *Acanthamoeba*.

Following the initial release of the *Acanthamoeba Rns* sequence in 1986, there was a lag in the analysis of succeeding isolates. Partial *Rns* sequences, suggesting that substantial variation existed within *Acanthamoeba*, were reported in 1990 [56]. A single full-length sequence for *A. palestinensis* CCAP 1547/1 (GenBank accession L09599) was deposited in the DNA databases in 1993, but not directly referred to in any early publication. It was not until 1994 that the situation began to change, with an increase in the number of available *Rns* sequences. A doctoral project by Rebecca Gast (in the Byers/Fuerst lab at OSU) involved the acquisition of almost complete *Rns* sequences from 18 strains of *Acanthamoeba* [57], which were deposited into the DNA databases. The paper that resulted from these analyses provided the foundation for the manner in which strains of *Acanthamoeba* are now routinely classified, the use of the concept of sequence types [58].

Sequence types were defined when an analysis showed substantial differences between the *Rns* sequences of some of the 18 strains of *Acanthamoeba*. In the initial paper [58], sequence types were defined as following: “The types are defined as sequences or groups of sequences that differ from all other sequences by at least 6%, have a minimum of 134 base differences, or an evolutionary distance greater than 0.8% in the current dataset.” What was not dealt with adequately in this initial definition was the fact that percent sequence difference (or the inverse, sequence similarity) depends on a comparison of homologous sites between the sequences. Determination of site homology depends on the accuracy of the sequence alignments, and these are affected by two important observations: the majority of sequence differences were found to occur within the expansion segments of the *Acanthamoeba* 18S rRNA gene, and expansion segments are not of uniform size in all isolates. As a consequence, sequence alignment can be problematic. Some attempts have been made to standardize the location of sequence differences within the secondary structure of the 18S rRNA, but this continues to provide a challenge to any definition that depends on comparisons of homologous sites. Consequently, the definition of a sequence type must be viewed with some flexibility as we have argued previously [59].

In the Gast et al. 1996 paper [58], sequences were available from isolates assigned to five of the “species” of *Acanthamoeba*: *A. castellanii, A. polyphaga, A. rhysodes, A. griffini and A. palestinensis*. The assumption made in defining sequence types is that sequence divergence represents evolutionary divergence. Our initial paper defined four sequence types. The distribution of sequences among the four types was far from uniform. Among the 18 strains analyzed, 15 of them were assigned to a single sequence type, T4, while the remaining three sequence types were represented by single isolates. Three species, *A. castellanii*, *A. polyphaga* and *A. rhysodes*, were contained within sequence type T4. Sequence type T3 was represented by a strain identified to *A. griffini*, while sequence type T2 was represented by a strain assigned to *A. palestinensis*. The single sequence type T1 isolate had been assigned to *A. castellanii*. Thus, from its first appearance, a sequence type can contain multiple nominal species, and a single nominal species can contain isolates from different sequence types. Again, the question is raised, what does a species of *Acanthamoeba* represent?

### 3.3. Increase in the Number of Sequence Types

Following the publication of our 1996 paper defining four sequence types, a small number of almost complete *Acanthamoeba* sequences were deposited in the DNA databases. A major expansion of sequences and sequence types occurred with the publication of our follow-up study [55]. In 1998, we reported an analysis of the almost complete *Rns* sequences from an additional 35 isolates, and combined them with our initial 18 strains. The number of sequence types was expanded by eight new types, T5–T12. Six of the new types (T6, T7, T8, T9, T10 and T12) were each represented by single isolates. Sequence type T1, remained with only a single strain. Additional strains were added into sequence types T2 and T3. Ten additional isolates were added to T4. Twelve isolates were assigned to sequence type T5, while two isolates were assigned to sequence type T11.

As had been seen in our initial study, multiple species occur within several sequence types; isolates from six species were assigned to T4; three species were represented by the three isolates in T2; three species occurred in the four T3 isolates; and, two species in the two T11 isolates. Isolates assigned to a specific nominal species were found in multiple sequence types. Nominal species *A. castellanii, A. polyphaga, A. palestinensis, A. culbertsoni* and *A. hatchetii* each occurred in more than one sequence type. In the 1998 paper [55], it was noted that the sequence differentiation between any two isolates assigned within a particular sequence type was always less than 5%. This value was not intended to be a fixed measure, but rather indicated an observation of our decisions to categorize isolates.

Following our proposal of sequence types [55,58], the utility of using this terminology to define the identity of new isolates began to be understood, and other labs began to add to the number of sequence types. In a study of the bacterial endosymbionts carried by *Acanthamoeba*, sequence type T13 was defined [60]. That same paper initially defined a strain as representing sequence type T14, but that was retracted, in part by the difficulty of defining sequence divergence in the presence of ambiguous alignments. Two years later, a new and different T14 was defined in two isolates from Pakistan [61]. This sequence type represents one of rarest forms that has been seen.

The descriptions of both sequence types T13 and T14 were based upon “almost complete” sequences of the 18S rRNA gene. Here, we are using the term “almost complete” to indicate that the sequences span almost all of the length of the gene. The original sequence of the *Rns* gene from the Neff strain was 2303 nucleotide in length [40]. The sequences in the original two papers dealing with sequence types ranged from 2224 to 3012 bp in length. In our study in 1998 [55], the nine sequences with length exceeding 2700 bases all contained self-splicing introns inserted into the gene, ranging from 488 to 699 bases in size. When the introns are removed, the length of each of the remaining sequences was 2293 or 2294 nucleotides. While only a single sequence from all isolates placed into either morphological *Acanthamoeba* Groups II and III exceeded 2300 bases, all three group I isolates exceeded 2500 bases, with the sequence obtained from the *A. astronyxis* isolate (ATCC 30137) being 2682 nucleotides in length.

In our subsequent analyses, we considered any sequence that was deposited in the databases that exceeded 2000 bases in length, excluding introns, to be viewed as “almost complete,” since these sequences would include all of the expansion segments of the gene and contain the regions of greatest variability and potential divergence between isolates. The definition of divergence between sequence types was thus based upon such “almost complete” sequences. This affects our consideration of subsequent descriptions of new sequence types.

In 2003, the partial sequences of a set of isolates identified morphologically as *A. jacobsi* were reported [62]. These sequences were similar to each other, but divergent from previously reported sequences associated with other species of *Acanthamoeba*. The sequences ranged from 1443 to 1475 bp in length, containing approximately the 5′ two-thirds of the gene sequence, and missing several of the expansion segments. Given the patterns of divergence from known sequence types, the designation of a new type was likely correct, but it took until 2017 for a complete 18S rRNA gene sequence from *A. jacobsi* to be obtained [63]. This confirmed the authenticity of type T15. One reason for the difficulty in obtaining a complete sequence became clear with the discovery that one of the *A. jacobsi* sequences included a 588 bp intron in a position that would have interfered with the PCR amplification of internal fragments of the gene in many isolates [63].

The identification of the T16 sequence type illustrates problems that can occur when there is not a neutral arbiter to evaluate sequence status. In 2009, two groups submitted manuscripts describing a new sequence type T16 [64,65]. Unfortunately, the two T16 proposals described isolates that were very different from each other. The data provided by Corsaro and Venditti involved an almost complete *Rns* gene sequence (*Acanthamoeba* sp. cvX, GenBank acc. # GQ380408), while the data from Lanocha et al. involved sequences slightly greater than 800 bases in length, starting approximately 600 bases into the gene. Keeping in mind our admonition that sequence types should be based on almost complete sequences, the T16 label should be applied to the taxa described by Corsaro and Venditti (2010), defining a new type that was a sister clade to the T13 sequence type. Interestingly, a highly similar, almost complete sequence had been deposited in 2001 (*Acanthamoeba* sp. U/H-C1, GenBank acc. #AY026245), the product of a doctoral thesis whose results were not subsequently published [66].

Despite the fact that they represented only partial sequences, analyses of the results of Lanocha et al. (2009) suggested that those sequences did represent a distinct group that might show closest sequence similarity to sequences assigned to T4. Further discussion of these findings is given below.

Additional almost complete sequences appeared over the next few years defining sequence types T17 [67], and T18 [46]. Both sequence types were found to be group I taxa. The description of T18 was the first since 2000 to link *Rns* sequence with the definition of the new species, *A. byersi*. In 2014, a new group III form was identified as sequence type T19 through an almost complete sequence of the 18S rRNA gene [68]. Sequence type T19 was subsequently tied to the description of the new species, *A. micheli* [47].

The correct designation of the sequences reported by Lanocha et al. (2009) [65] began to be clarified next. In 2006, a series of partial sequences were deposited in the DNA databases from a study of *Acanthamoeba* isolated from a lethal infection of a keel-billed toucan (*Ramphastos sulfuratus*) [69]. These sequences were all approximately 430 bp in length, overlapping the most variable expansion segment of the 18S rRNA gene. The sequences were originally identified as most similar to, but divergent from, sequences assigned to sequence type T4. Given the divergent nature of the sequences, a reanalysis was subsequently performed to examine almost complete sequences, and these were found to represent a new sequence type, now designated T20 [59]. Also in 2016, the Polish group published additional, now almost complete sequences, closely associated with the new T20 [70]. The Polish sequences were found to be similar, but not identical to the expanded sequences of the OSU lab, but consistent with variation within a sequence type.

The last clearly identified sequence type, T21, represented a major morphological break from other *Acanthamoeba*. An examination of the inclusive nature of the family Acanthamoebidae was reported in 2016 [48]. “*Protostelium*” *pyriformis* was the name previously applied to a sporocarpic amoeba. Sporocarpic amoebas are amoebae that individually form a walled, dormant propagule elevated by a non-cellular stalk, not a characteristic of any previously identified member of *Acanthamoeba*. Nevertheless, molecular analyses involving a number of genes, including the 18S rRNA gene, indicate that this amoeba is a member of *Acanthamoeba*. Renamed *Acanthamoeba pyriformis*, it appears to share a most recent common ancestor with the joint lineage leading to group II and III, after that joint lineage diverged from the line leading to the contemporary group I acanthamoebae. The almost complete 18S rRNA gene of *A. pyriformis* shares the expansion segments seen in other members of *Acanthamoeba,* but certainly shows significant sequence divergence that exemplifies a new sequence type.

One sequence type remains to be discussed. This type is poorly understood, and should continue to remain provisional. The type is represented by a insufficiently understood set of sequences that represent the results of a whole-genome shotgun sequencing project that purported to examine *Acanthamoeba royreba* strain Oak Ridge [AC-023] (ATCC 30884). This ATCC isolate has been well studied previously, and was even included in the original investigation that resulted in the proposal of sequence types [58]. All earlier analyses of this ATCC isolate have indicated that it is a member of sequence type T4. When the 18S rRNA sequence was extracted from SRA and whole-genome sequence (WGS) files available for the genome project in GenBank (WGS CDEZ00000000), the sequence clearly represents a member of *Acanthamoeba,* and contains the expansion segments characteristic of *Acanthamoeba*. The genome-derived *Rns* can be placed phylogenetically closest to the T15 and T19 types, not T4. Further, the sequence is unlike any reported previously, and is sufficiently divergent to warrant being considered to represent a new sequence type, designated T22. The sequence is listed in a Supplemental File, Table S1. It could be argued that aspects of accumulating sequences in the WGS process might have resulted in an erroneous *Rns* result. However, we have used those same files to extract the entire mitochondrial genome sequence, and examination of sequences of each of the genes in the mt-genome suggest a distinct taxa, and one that is not a part of sequence type T4.

### 3.4. “Almost Complete” and Partial 18S rRNA Gene Sequences in the Study of Acanthamoeba

**Figure 1:**
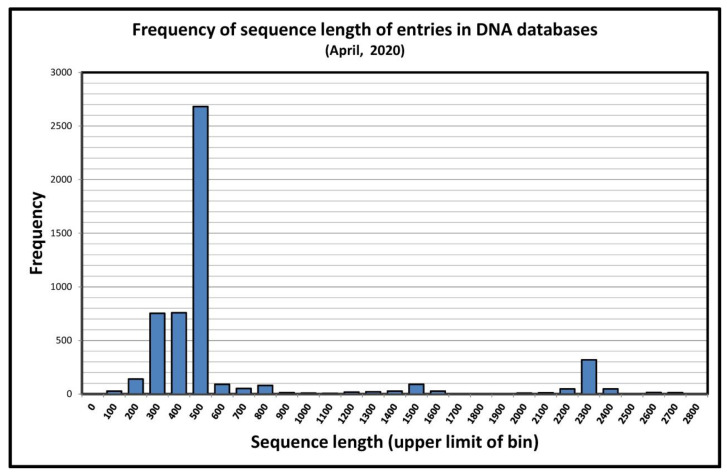
Distribution of sequence length for *Acanthamoeba* 18S rRNA gene sequences deposited in the international DNA databases or provided by researchers.

Mention has been made of partial sequences of the *Rns* gene that were important in the determination of several new sequence types. In the study of *Acanthamoeba* using the 18S rRNA gene as a diagnostic target, the importance of partial sequences has been overwhelming. In April 2020, our database (http://u.osu.edu/acanthamoeba) had recorded over 5500 *Rns* sequences that had been deposited in the international DNA databases, or had been provided directly to us by researchers. Sequences that exceeded 2000 bases in length represented only 447 of these sequences. Partial sequences ranged in size from 47 to 1976 nucleotides in length. The distribution of the size of sequences that have been determined is not uniform over this range of sizes. The distribution of the sizes of *Rns* sequences in our database is shown in Figure 1. More than 3900 partial sequences fall into a range between 210 and 480 nucleotides in length. Even here there is heterogeneity, with 638 sequences between 210 and 260 nucleotides in length, while a second group of 2804 isolates fall in the range 390–470 nucleotides. Why are these sizes so popular?

The reason for the popularity of fragments of a particular size to characterize an isolate traces to a paper that suggested that subgenic fragments could be used to study *Acanthamoeba* [71]. The paper, which has been cited almost 500 times, provides information on PCR primers that can generate several different subgenic fragments. One fragment was designated ASA.S1, but is often referred to as JDP1-JDP2, referring to the PCR primers used to amplify the fragment. The use of these primers would generate a fragment slightly greater than 450 basepairs. This fragment can be sequenced directly, or one can use an additional sequencing primer internal of the ASA.S1 fragment, which will yield a sequence referred to as DF3, with a size of 200+ basepairs [72].

**Figure 2:**
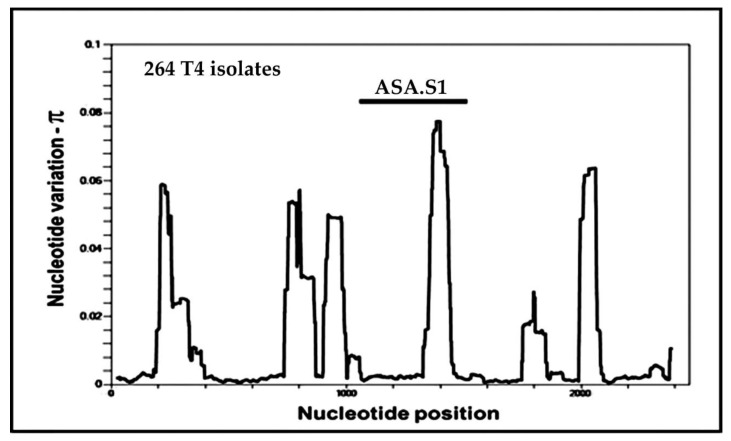
Values of nucleotide diversity (π) in a sliding window of 25 nucleotides across “almost complete” *Rns* sequences from 264 *Acanthamoeba* T4 isolates.

Subsequent analysis has shown that the ASA.S1 fragment overlaps the most variable portion of the 18S rRNA gene sequence of *Acanthamoeba*. To illustrate this point, the distribution of genetic variation across the length of *Acanthamoeba Rns* sequences is shown in Figure 2. For a set of 264 almost complete *Rns* sequences from *Acanthamoeba* sequence type T4 isolates, nucleotide diversity (designated π) [73] was calculated in a sliding window of 25 bases across the length of the gene. Several regions of high diversity are indicated, each associated with a region that includes expansion segments of the gene. The region of highest gene diversity corresponds to the ASA.S1 segment of the gene. Examination of the set of 447 almost complete sequences indicates that the use of the ASA.S1 fragment to classify isolates would group isolates correctly by sequence type based on the entire gene sequence. Although not universally utilized, the ASA.S1 fragment has become the most targeted genetic segment in survey studies of isolates of *Acanthamoeba*.

Much variation clearly exists within the ASA.S1 region. Many isolates with partial *Rns* sequences have been examined only for this region. Have we missed significant aspects of variation when we fail to use almost complete sequences to classify sequence types? With this question in mind, we will now further refine our analysis of variation.

## 4. Alleles in the ASA.S1 Region of the *Acanthamoeba* 18S rRNA Gene

### 4.1. The Genesis of “Alleles” in Acanthamoeba

In the early 2000s, researchers began to increasingly use 18S rRNA gene sequences to identify and characterize isolates of *Acanthamoeba*. The use of partial sequences began to be a preferred approach following the demonstration that segments of the gene could accurately identify the various sequence types [71]. By 2001, it had become evident that the majority of isolates in clinical or environmental studies were classified into sequence type T4 [55,71,74]. It was realized that clustering of cases of *Acanthamoeba* keratitis (AK), or tracking of strains in the environment might be possible by examining subsequences within the ASA.S1 fragment [71,72].

The first attempt to examine subsequences of ASA.S1 to identify individual isolates occurred in a study of clinical and environmental isolates of *Acanthamoeba* in Hong Kong [72]. In the study, 17 T4 isolates and 5 T3 isolates were obtained. When the sequence within a portion of the DF3 subregion of ASA.S1 was examined, 10 unique sequences were found in the T4 isolates and 5 different sequences were obtained in the T3 sequences. The allele segment was defined as existing between specific conserved segments of the region, ACCACCAT on the 5′ flank, and TGGCAC on the 3′ flank of the segment. These sequences were designated as alleles, and designated T4/1 through T4/10 and T3/1 through T3/5. The allele segments in T4 isolates ranged in size from 54 to 64 bases, while the T3 alleles were 58 to 65 bases in length.

Each of the T3 isolates possessed their own unique alleles in the Hong Kong samples [72]. This was not the case for alleles of T4 alleles. One allele occurred in three isolates, while five alleles occurred in two isolates. The samples were designated by location or patient. For some of the locations or patients, multiple samples were collected from different sampling sites, for example corneal scrape, contact lens or contact lens case, including left or right case, or from faucets or other water sources in the homes of patients. For four of the T4 alleles, the multiple occurrence of the allele was linked with samples taken from a single patient. However, distinct alleles were found in different isolates from three of the patients, one patient with different T4 isolate alleles, one patient with different T3 isolates and one patient with different T4 alleles and a T3 allele. None of the alleles seen in seven environmental isolates appeared in any “clinical” material from a patient (eye, contact lens or case). However, two environmental isolates from different locations carried the identical allele. Clearly, alleles gave new insights into how to examine *Acanthamoeba* in both the clinic and the environment.

The next attempt to extend the allele concept involved a study from the University of Miami of isolates from AK patients [75]. The study was carried out by an alumna of the Byers/Fuerst lab, Dolena Ledee. Alleles were defined mostly based on the region previously identified in studies in Hong Kong [72]. However, in Miami, alleles were cut off five bases from the ends defined in Hong Kong. For global comparisons, we have appended the last five nucleotides to the allele definitions given in Ledee et al. (2009) [75]. In Miami, investigators observed two of the alleles seen in Hong Kong (T4/2 and T4/6). In addition, eleven new alleles were observed, designated T4/11–T4/21. Just as was observed in Hong Kong, in some cases (six patients), all material associated with a particular patient were found to have a consistent allele. However one patient showed different alleles in isolates from two parts of the lens case. The size range of T4 alleles was extended by allele T4/11 which was 69 bases in length.

It is interesting to note that nine of the first 21 alleles identified had already been observed in the first 53 *Acanthamoeba* isolates that had been studied for the definition of sequence types [55,58], with six of the alleles observed in 1996 (T4/8; T4/9 in 3 strains; T4/10 in 2 strains; T4/16, T4/20 and T4/21), while three additional alleles were observed in 1998 (T4/6 in two isolates; T4/12 and T4/13). The first two studies on alleles reported 21 different alleles when the DF3 region of ASA.S1 was studied. Where would this approach lead?

### 4.2. The Early Expansion of “Alleles” in Acanthamoeba Type T4

Unfortunately, with no central clearing mechanism to keep track of new alleles, confusion began to occur. In 2010, two studies appeared that defined new allele types, but did so independently and without coordination, resulting in identical designations for different allele sequences [76,77].

Abe and Kimata [76] studied seven Japanese isolates from three AK patients. They observed consistent genotypes from multiple isolates of each patient. Their isolates all contained either of two “new” alleles, labeled T4/22 (two isolates from one patient) and T4/23 (three isolates from one patient and two from another). The T4/22 allele matched the sequence of one of the isolates originally reported in 1996, *A*. sp. Rawdon ATCC 50497 [58], as well as matching a number of isolates that had previously been reported by several groups.

The second report studied 14 isolates from China, each from a different AK patient [77]. Two of the isolates represented sequences already seen (T4/6 and T4/13). The remaining isolates carried seven allele sequences not previously described, labeled as T4/22–T4/28. Obviously T4/22 and T4/23 duplicated the numbers from Japan, but they represented different allele sequences. Two of the alleles were observed more than once (three occurrences of T4/24, four occurrences of T4/25). The other alleles occurred only once in the study.

The same type of duplication of an allele designation occurred in three papers appearing in 2013. First, in a study of Brazilian isolates [78], investigators found one AK isolate containing an apparent new sequence and designated the allele sequence as T4/29, acknowledging the seven alleles described in 2010 from China [77]. Note that since its original occurrence, the allele sequence represented by this Brazilian isolate has not been observed in any other isolate among the more than 3750 T4 isolates in our database. A second study, in Spain, examined 38 *Acanthamoeba* isolates obtained from drinking water treatment plants [79]. In studies of the DF3 region of ASA.S1, nine different alleles of type T4 were observed, including four novel alleles. Being apparently unaware of the alleles described in 2010 [76,77], the new alleles were designated T4/22–T4/25, further confusing the numbering system. None of the new alleles had been reported previously. The T4/22 allele of this study was observed in five independent isolates, while the other alleles occurred only once. The study found multiple isolates carrying allele T4/1 (3 isolates), T4/8 (23 isolates) and T4/13 (2 isolates). There were also single isolates found carrying alleles T4/9 or T4/12. No isolates of T3 were reported from the study.

Three different alleles now existed that were assigned to the designations T4/22 and T4/23 and two different alleles for T4/24 and T4/25. Confusion was starting to expand, but more now occurred. A third separate 2013 study, in France [80], which was submitted on the exact date that the Spanish study had been submitted, examined twenty *Acanthamoeba* isolates. Results showed 16 T4 isolates and 2 T3 isolates, along with single isolates of T2 and T5. The T3 isolates carried alleles (T3/3 and T3/4) that had been observed previously. Among the T4 isolates, four previous alleles were found, including four isolates with T4/7, single examples of T4/8 and T4/10, and two isolates carrying the T4/22 allele as defined by Abe and Kimata [76] which was initially designated in the French paper as T4/22b. Given the duplication of labels T4/22 and T4/23 that the French group was aware of, it was suggested that the Japanese alleles be relabeled as T4/29 and T4/30. However, they were unaware of the prior designation in an earlier paper of T4/29 [78], now resulting in a duplication of T4/29. The remaining T4 isolates from the French study contained three additional new alleles, T4/32, occurring in three isolates, and T4/33 and T4/34, each occurring once. It turned out that the allele designated T4/34 was identical in sequence to the Spanish T4/24 allele, fostering further difficulties. Confusion had potentially increased to three versions of T4/22, T4/23, and T4/24, and two versions of T4/25 and T4/29. As a final part of their studies, further considerations of allele designations beyond their own isolates were taken up in a supplemental table of the French study, but that supplemental table appears to have some problems, which will be considered below, and ultimately lead to the conclusion that the extension of labels for alleles within the ASA.S1 region must be approached with care.

One important aspect of the French study [80] was their examination of variation in regions other than the ASA.S1. They investigated variation in the V4 hypervariable region, which extends from approximately bases 700–1000 of the gene, as shown in Figure 2. By comparing the V4 and DF3 variable regions, they found *Acanthamoeba* isolates with the same DF3 sequence were not necessarily identical throughout the gene. It is certainly not surprising that the ASA.S1 region would not solely identify an isolate, and information from other regions would certainly be useful. However, ASA.S1 has proven a very useful diagnostic fragment in *Acanthamoeba*. The overwhelming proportion of *Acanthamoeba* isolates have been studied including only the ASA.S1 region, or a subportion of this region such as DF3. Much smaller numbers of isolates have been studied for other regions within the gene. While calling for analysis of additional regions of the *Rns* gene is worthwhile, it is unlikely that a call for longer sequences to characterize a sample would be widely followed, since the use of parts of the ASA.S1 region to provide a small diagnostic tag has proven very successful in identifying the presence of *Acanthamoeba*. For those interested in the absolute identification of isolates, complete or almost complete sequences should be viewed as the gold standard. Even then, the question can be raised of whether a single gene such as the *Rns* gene is sufficient to characterize an organism’s genome.

Since 2013, we know of no publication that has designated new alleles. However, beginning in July 2015, allele types have been enumerated and updated on our *Acanthamoeba* website [53].

### 4.3. Enhancing the Concept of “Alleles” in Acanthamoeba in T4

With confusion and duplication of allele numbers becoming extensive, some ordered approach appeared to be required before identifying any new “alleles.” There are several factors that we consider important for the classification of alleles in 2020, with more than 5300 *Acanthamoeba Rns* sequences to evaluate. First, sequences are disregarded if they do not completely overlap the ASA.S1 region that is used to define alleles. If the DNA sequence that was deposited contains any ambiguous nucleotides at any sites within the allele region, it will also be disregarded. Finally, if a sequence of the allele region is found to occur in only a single isolate, it will not be numbered, at least until another matching isolate is reported. With more than 3700 T4 isolates and more than 1500 isolates categorized into other non-T4 sequence types in 2020, our aim is to identify groups of isolates that may be related, rather than simply to gather a list of all possible alleles.

We have opted to retain in our expanded list any allele designation that was identified prior to 2015, regardless of whether the allele sequence occurred in multiple isolates. Allele numbering ambiguities were dealt with as follows: (1) the first 21 alleles would retain their identifiers (T4/01 to T4/21) [72,75]; (2) alleles identified in 2010 or 2013 would be labeled as defined in the original papers, but with a prefix identifying the author source of the label. Thus, alleles from Japan [76] would be labeled AKT4/22 and AKT4/23, alleles from China [77] would be labeled ZT4/22 to ZT4/28, the allele from Brazil [78] is labeled DT4/29, and the alleles from Spain [79] are labeled MT4/22 to MT4/25. Finally, the new alleles observed in the study from France [80] are labeled RT4/31 to RT4/34. Note that RT4/34 and MT4/24 are identical in sequence; since both papers were submitted on the identical date, but, since the French paper was accepted earlier, we assign any isolates with this sequence to allele RT4/34.

In their paper, Risler, Coupat-Goutaland and Pélandakis [80] presented an additional analysis based mainly on many of the isolates with almost complete *Rns* sequences that were in the DNA databases. This secondary study resulted in a list of additional alleles that was provided in a supplemental table, but not in the paper itself. The supplemental table proposed a series of additional alleles labeled T4/35–T4/73, but contains a number of problems and inconsistencies when examined closely. First, the authors were unaware of other publications during 2013 that also proposed new alleles. Second, one of the proposed allele sequences duplicates an allele from the original group of 21 alleles [72,75]. Third, six other of the proposed alleles do not match the sequence from the isolate listed in Genbank that was used to generate them, and do not seem to exist within any other isolate in the DNA databases, neither in the international databases such as GenBank nor in our own *Rns* database that includes additional sequences never deposited in GenBank. Finally, 14 of the additional proposed alleles occur in only a single isolate in the DNA databases. As mentioned above, we strongly argue that alleles should be given a label only if they occur in multiple isolates in the databases. It is easy to determine, by using BLAST [81], whether a new isolate contains an allele that has been seen before. These problematic labels are scattered throughout the table, and so we started anew in 2015.

One final additional problem existed in the allele labels that were used by Risler, Coupat-Goutaland and Pélandakis [80]. Examining the sequences from isolates in their study that were deposited in GenBank, we found that several do not match the allele sequence that is listed in Table 1 of their paper. This led to an assumption on our part about the sequence of both the RT4/32 and RT4/33 alleles. In 2015, when we started to expand our studies of alleles, we randomly used the sequence from isolate AcL-JN15 (GenBank accession HF930505), labeled as RT4/32 in the French paper’s Table 1, to represent the sequence of the RT4/32 allele. Similarly, to represent the RT4/33 allele we used the sequence from French isolate AcL-LA16 (acc # HF930509). Only several years later did we realize that the allele from AcL-JN15 does not match the sequence given in the supplemental table of the paper, and in fact occurs only in AcL-JN15 among isolates in the databases. Similarly, the sequence given in the supplemental table does not match AcL-LA16, nor does it match the sequence for *A. polyphaga* Nagington (acc #AF019062), which is used as the exemplar for RT4/32 in the supplemental table. Nevertheless, AcL-LA16 and *A. polyphaga* Nagington do match each other for allele sequence. In the case of allele RT4/33, the sequence that is given in the supplemental table does occur in two isolates in the databases unrelated to the French study. We apologize to the French group, but we have since attached a new label to the sequence that was listed as RT4/32 in the supplemental table, a sequence which did match two other isolates from the French study. Similarly the sequence for RT4/33 from the supplemental table was also given a new label. So in the end, duplication lingered. The sequences designated RT4/32 and RT4/33 in our later table are the sequences taken from the French AK isolates indicated above.

### 4.4. Extending the Analysis of “Alleles” in Acanthamoeba T4 Post-2015

Since our website launched in 2015 [53], we have monitored new submissions of 18S rRNA gene sequences to the international databases and incorporated information about any novel alleles that occur in multiple isolates. In addition, all isolates are characterized for the alleles they carried. Initially, we applied this approach to isolates from sequence types T3 and T4. Subsequently, we extended this to isolates from sequence type T5, T11 and T15—all of which are now represented by more than 100 deposited sequences. When a publication appeared in which sequencing of the *Rns* gene was reported, but sequences were not deposited, we have tried to contact the authors, and encouraged them to share their data, either by depositing the sequences to the international databases or sharing the sequences with us. As a result, we have information on more than 5300 18S rRNA gene sequences from *Acanthamoeba*, including almost 3800 isolates classified within sequence type T4.

When we began to extend the earlier analyses of alleles, 38 alleles had been described for sequence type T4 (accounting for the duplication represented by MT4/24–RT4/34). The frequency of isolates in March 2020 observed carrying these early alleles is given in Table 5.

We began an expanded list in 2015, beginning with an allele labeled OT4/39 (the O indicating OSU, The Ohio State University), and adding new OT4/## alleles from that point. By March 2020, we had reached OT4/158, thus adding 120 described alleles to the list. As described previously, the criterion used to define a new allele is that the allele must occur in more than a single isolate in the database. Note that among the first 38 alleles that were defined, seven have been observed in only a single isolate, and one (M4/24) does not match any isolate (because of an error in the initial report). The sequences within ASA.S1 that characterize these alleles are provided in Supplemental Table S2. A pdf version of the list, updated whenever a new allele is identified, is available on our website [82].

By chance, OT4/39, the first new allele, unintentionally matched the sequence of RT4/32 given in the supplemental table of Risler, et al. [80]. This allele is of great importance, since it is the allele that is carried by the type isolate for the genus *Acanthamoeba* (*A. castellanii* ATCC 30011 or CCAP 1501/10). As mentioned, there are numerous inconsistencies in sequences in the supplemental table of Risler, Coupat-Goutaland and Pélandakis. The sequence that we had used for RT4/32 previous to 2015 was based on that found for the isolate AcL-JN15, described as carrying RT4/32 in Table 1 from their paper [80] a sequence inconsistent with the sequence of RT4/32 in their supplemental table. Because of our use of the OT4/39 label since 2015, we are retaining this label for isolates that carry the allele, and have not used the RT4/32 label defined in the supplemental table of Risler et al. [80].

The frequencies of each of the 120 post-2015 alleles defined at the time of submission are provided in Table 6, and sequences are given in Supplemental Table S2, and have been previously defined on our web site [82]. Table 5 and Table 6 provide numbers tabulated from 2914 isolates categorized as *Acanthamoeba* sequence type T4. Alleles occurring in isolates of sequence type T4 ranged in size from 52 to 72 nucleotides in length. However, there are additional T4 isolates deposited in the databases that are not included in this tally. For example, the sequences for 541 T4 isolates in the international databases do not overlap the region of the alleles, either partially or completely.

Another class of isolates whose sequences exist in the databases but that were excluded from the tables includes a group of 98 isolates containing multiple sites that were recorded as ambiguous reads within the allele region. A sample could exhibit either single or multiple ambiguous sites. For example, some sites occur with either Ys or Rs indicating that the base at a site might be either cytosine or thymine, or either guanine or adenine. Other combinations of bases also occur.

Such ambiguous sequencing reads could have several sources. First, they could simply be samples for which the read was imperfect. Second, they could embody cases in which the DNA sample was obtained from an unrecognized mixture of isolates, i.e., a sample that included two or more *Acanthamoeba* strains that had not been adequately separated from each other during culture. Unrecognized mixtures would often result in “alleles” that look different from any previous sequence deposited in the databases. An unrecognized mixture whose “sequence” was deposited in the databases would often show multiple nucleotide differences from all other samples which might possibly also include some ambiguous sites, and might often include in/del differences when compared to all other isolates. Multiple in/del sites predicted by a BLAST comparison [81] with other sequences in the database can easily occur if the unrecognized mixed sample contains the DNA from two isolates whose alleles differ in size.

Unrecognized mixtures of DNA from different amoebae are not the only possible source of sequences that contain single or multiple ambiguous sites. Another source is intracellular polymorphism. In most eukaryotes, including *Acanthamoeba*, a single precursor RNA is transcribed that contains the small ribosomal subunit RNA (18S) together with the large ribosomal subunit RNAs (5.8S and 28S). These RNAs are all processed from the single precursor following transcription from the rRNA cistron. The transcriptional unit in turn forms part of a tandem repeat, which can occur on one or more chromosomes. Estimates have been made that there are at least 60 copies of the repeat unit in tandem arrays within the genome of *Acanthamoeba* [83,84]. A process known as concerted evolution acts to keep the copies of the gene identical, or very similar [85]. Concerted evolution is the non-independent evolution of repetitive DNA sequences, often attributed to recombinational processes within the genome, resulting in a sequence similarity of repeating units that is greater within than among species [86,87]. As evolutionary time proceeds, the same DNA sequence is maintained in each of the multiple cistrons of an array, even though differences between the arrays of divergent species are allowed to accumulate differences. However, this process is imperfect. Since divergence between evolutionary units does occur, there must be a time when polymorphism exists between some of the members of the array. This type of polymorphism has been seen in *Acanthamoeba* [84,88]. Polymorphism for the 18S rRNA gene was defined early in our studies of sequence types [55], in which 7 of 53 strains of *Acanthamoeba* were found to contain multiple *Rns* sequences. One isolate, TIO:H30, appeared very complex, and was subsequently found to contain 3 alleles [88].

**Figure 3:**
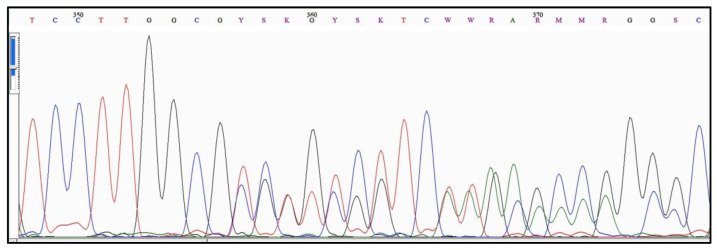
Example of a polymorphic sample from a portion of the allele region of a T4 isolate. The overall sequence represents two alleles within a single isolate of *Acanthamoeba.* The electropherogram shows multiple peaks at numerous sites within the sequence. Knowledge of potential allele sequences from *Acanthamoeba* allows the identification of both alleles present in the isolate.

An example of the type of sequencing output that sometimes results from polymorphism for the allele region of the *Acanthamoeba Rns* is shown in Figure 3. The sample is from a single isolate that has been subcultured to try to ensure that it represents a single initial amoeba.

The region shown in Figure 3 begins 10 bases 3′ from the start of an allelic region. This isolate contains a polymorphism involving the presence of two different alleles within the sample. A comparison of the DNA read from the electropherogram shown in Figure 3 and the two alleles carried by the sample is given in Table 7. The allele designations listed in Table 7 are based on the sequence of the entire allele—only part of which is shown in Figure 3. Note that an isolate containing this combination of alleles has occurred at least twice in the databases. Examination of Table 7 indicates that a sequence corresponding to allele T4/18, can be subtracted from the DNA read, leaving a sequence that corresponds to a second allele, T4/134. Both alleles have occurred singly in multiple samples in the DNA databases. Comparison of the complete sequences of alleles T4/18 and T4/134 shows that they differ by at least two in/del events. If a careful analysis was not performed of the region where multiple peaks occur in the electropherogram, and the sequence was deposited as directly read from the electropherogram, the sequence would be classified as ambiguous, and ultimately viewed by us as probably a mixed sample. It is a mixed sample, but one due to an intracellular polymorphism.

We suspect that at least 65 isolates among those that have been deposited in the databases actually represent the sequences obtained from either mixed samples of isolates or significant polymorphism within a sequencing sample. This is probably an underestimate. These 65 samples have been classified as carrying unique singleton sequences, with no ambiguous bases in the allele region. There are also a number of additional sequences that occur more than once in the databases, and which we suspect also represent mixed samples. Since they occur in multiple samples, we have included them in our allele counts. Unfortunately, we do not have access to the electropherograms from any of these samples. With access to the electropherograms of these samples, we feel that most would be correctly classified as mixed, and that the alleles present in the sample could be identified.

Finally, there are 298 isolates deposited in the DNA databases whose sequence in the allele region is unique. Some of these may be mixed samples or samples with polymorphisms carrying alleles that are close in sequence, but which have not been scored for an ambiguous nucleotide. Many of the 298, however, probably represent true singleton alleles.

### 4.5. Frequency of “Alleles” in Acanthamoeba Sequence Type T4 Post-2015

**Figure 4:**
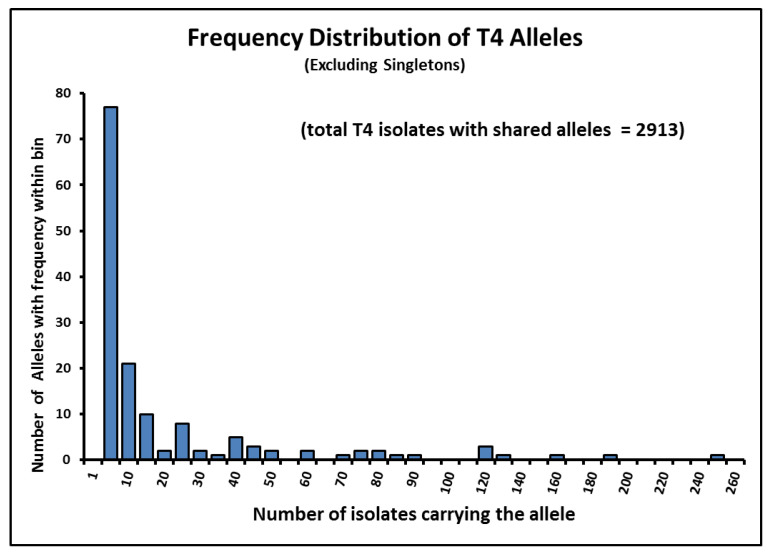
Allele frequency distribution for 151 alleles that occur in multiple isolates of sequence type T4 within the DNA databases.

Given the information summarized in Table 1 and Table 2, we can begin to ask about the patterns that can be observed for the alleles that occur in more than two isolates within sequence type T4. The two tables provide information about 2914 isolates, partitioned into 158 allele classes. As mentioned, a number of the original 39 alleles have occurred only a single time within the database, and one sequence does not occur in any isolate. This leaves 151 alleles that have been observed in more than a single isolate. The allele frequency distribution of alleles occurring more than once in the DNA databases is shown in Figure 4.

Seven of the alleles occur in more than 100 deposited isolates. The most frequent allele was AKT4/22, occurring in more than 240 isolates. This allele was first observed in our original sequence type study [58], from the strain *A*. sp. Rawdon (BCM:88-2-37; ATCC 50497). It has been found in isolates classified as *A. polyphaga*, *A. lugdunensis*, *A. rhysodes*, and mostly in strains designated “*A*. species.” Five other strains deposited in culture collections also carry this allele. They include *A. lugdunensis* clone L3a (ATCC 50240), *A*. sp. CDC:V087 (ATCC PRA-81), *A.* sp. 17 strain 25-349-MX (ATCC 50670), *A. polyphaga* Linc Ap-1 (CCAP 1501/18), and as one of the alleles found in *A*. sp. TIO:H30 (ATCC 50726).

The second most frequent allele, found in 182 isolates, is the allele OT4/39, carried by the type strain for the genus *Acanthamoeba, A. castellanii* (Douglas) Page AC30 (ATCC 30011; CCAP 1501/10). The allele also occurs in a number of strains maintained in the culture collections, including *A. castellanii* (ATCC 30234) and *A. castellanii* (ATCC 50374)—both of which are subcultures of ATCC 30011—as well as in *A. castellanii*, strain Naginton 1974 (ATCC 30868; CCAP 1501/2g), *A*. sp. strain UWC6 (ATCC PRA-1), *A*. sp. strain UWC8 (ATCC PRA-2), *A*. sp. strain UWE2 (ATCC PRA-8), *A*. sp. strain CDC:V155 (ATCC PRA-82) and *A.* sp. strain CDC:V522 (NR-46473).

The only other allele found more than 150 times was allele T4/06, which occurred in two of the isolates studies in our second sequence type paper [55], *A.* sp. strain CEI:M95:5:27, Vazaldua (ATCC 50723) and *A.* sp. strain Diamond CDC (ATCC 50724), as well as in one other strain in the culture collections, *A. polyphaga* HN-3 (ATCC 30173).

Four other alleles, T4/02, T4/08, T4/13 and OT4/48, occur more than 100 times. Nine alleles were found to have between 51 and 100 occurrences, while 33 alleles occurred between 11 and 50 times. Rare alleles dominate the distribution, with 21 alleles occurring between 6 and 10 times, and 77 having only 2–5 copies. There were 46 alleles that occurred 2 times. Not shown on the graph are the 298+ isolates that occurred as singletons within the database.

### 4.6. Occurrence of Specific “Alleles” in Subtypes of Acanthamoeba Sequence Type T4

We have argued that the concept of sequence types does not capture the true diversity of genetic types within *Acanthamoeba* [89]. Examination of the almost complete sequences within sequence type T4 suggests that at least seven subgroups exist, which we have labeled T4A–T4F and T4Neff. Examination of defined alleles should provide understanding of the partitioning of genetic diversity within the most frequently encountered type within *Acanthamoeba*.

We have examined the pattern of allocation of alleles into the seven subtypes of T4 by first investigating how alleles are partitioned among the isolates for which almost complete *Rns* sequences are available. The results for these isolates are presented in Table 8. For these 404 isolates, each isolate can be placed into a subtype, by comparison with a set of sequences that we have identified that exemplify the sequence characteristics of each subtype [59,89]. For all isolates classified within a sequence subtype, we then determined whether the isolate carried one (or, in a few cases, two or three, when polymorphism had been determined to occur) of the 151 alleles that occur in multiple isolates in the DNA databases, or whether the isolate carried a singleton allele. No almost complete sequence had ambiguous reads in the allele region.

Aggregating alleles from all of the isolates carrying almost complete *Rns* sequences showed that these isolates, which represent approximately 13% of T4 isolates classified for alleles, include 56 of the 151 alleles from the databases. Although many of the isolates classified within each of the subtypes may carry the same allele, there is considerable variation that still exists within each subtype. However, summing the middle row of Table 9 will reveal a striking finding. The sum of the number of alleles within the subtypes is equal to the total number, 56, of alleles that exist within the entire set of almost complete T4 sequences. None of the alleles occurs in more than a single subtype. There is no allele that is shared between subtypes.

Table 9 provides similar data for the complete set of sequences, combining almost complete and partial sequences of the 18S rRNA, but excluding non-overlapping or ambiguous sequences.

Approximately 10% of isolates (299 of 3130 isolates classified for an allele) can be categorized as a singleton or unique allele. In the entire dataset for T4 isolates, there are 151 alleles found in multiple isolates. Summing the middle row of Table 5 (total = 151), illustrates, as was found for isolates with almost complete sequences, that no allele, even among the extended set of alleles and isolates, is shared between subtypes of type T4.

**Figure 5:**
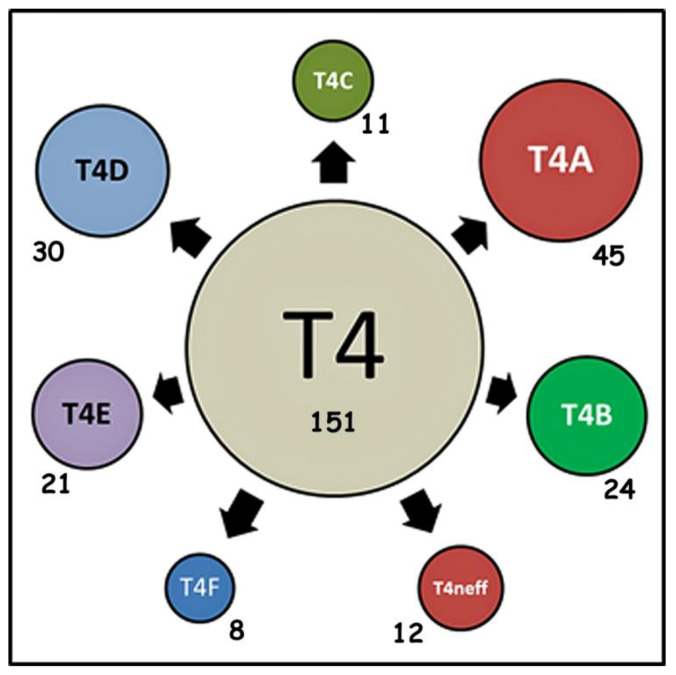
A representation of the separation of alleles into the seven subtypes of T4. Area in circles equivalent to the proportion of T4 isolates classified into subtypes. No allele is shared between subtypes. Numbers below circles indicate the number of alleles found in each T4 subtype.

The pattern of separation of alleles into subtypes is represented in Figure 5. In the figure, the assemblage of all alleles is shown to exist within the central unit, representing sequence type T4 as a whole. The common ancestor of all T4 isolates, essentially a precursor to the central unit then evolved into separate subtypes over time. This separation is indicated in Figure 5, where the area within the circle of each subtype in the figure is equivalent to the proportion of all T4 *Rns* sequences in the database.

As subtypes diverged from the common ancestor of all T4 isolates, alleles within a subtype would have evolved from sequences that existed within common ancestors of the two or more subtypes as they sequentially diverged. Alleles would become differentiated during evolution by mutations that independently occurred within the separate lineages leading to each subtype. Some of the ancestral sequences may still be retained, but only within one subtype. We have previously done various phylogenetic analyses of the sequences of the *Rns* gene in *Acanthamoeba*. None of the phylogenetic analyses using the almost complete *Rns* sequences produce a tree with bootstrap values of 100% on the branches separating each subtype. In contrast, the analysis of alleles indicates a clear and complete separation of alleles into subtypes. This strongly indicates that the subtypes represent independent evolutionary units, but units that are difficult to define in a normal phylogenetic analysis because of the difficulty of adequately defining homologous sites within alleles. Further work on defining the evolutionary units represented by subtypes (i.e., whether they are “real” species) will depend on gathering additional genetic information beyond the single dataset represented by the 18S rRNA gene.

## 5. Alleles in the ASA.S1 Region of the 18S rRNA Gene in Non-T4 *Acanthamoeba* Sequence Types

We have also examined the variability of the *Rns* gene in other sequence types. In addition to sequence type T4, the DNA databases contain over 100 isolates for several other sequence types. These include T3, T5, T11 and T15. We have examined allele variability within these four sequence types. Sequence types T5 and T15 are the only sequence types that are currently each associated with a single species, *A. lenticulata* and *A. jacobsi*, respectively. There are also more than 300 isolates that are classified within the complex T2/6 supergroup. We have analyzed this supergroup for the first time for this paper.

### 5.1. Alleles in Acanthamoeba Sequence Type T3

At the time of submission, the *Rns* sequences for 316 isolates assigned to T3 had been deposited in the DNA databases. Of these, 40 isolates have been represented by almost complete sequences. When the distribution of genetic variability over the length of the gene is examined, a pattern very similar to that shown in Figure 2 was obtained, indicating that the region with greatest nucleotide variability (π) is the same ASA.S1 region that was identified in sequence type T4.

Five alleles had been identified for T3 isolates in our first study of alleles in *Acanthamoeba* [72]. The alleles, as described in our initial study, were defined to begin one nucleotide after the start of the T4 allele region. We have opted to add back that base, which was a conserved G, to standardize alleles in different sequence types. In our first study each T3 allele was found in a single isolate [72]. As far as we are aware, no other attempt has been made to categorize alleles in T3. Since 2015, we have enumerated alleles within sequence type T3 on our website [90]. The sequences identified with T3 alleles are given in Supplemental Table S3. Alleles found in multiple T3 isolates have a size range from 59 to 66 nucleotides within the allele region.

When examining allelic variation among the 40 almost complete sequences, five alleles were observed, including three of the alleles that we originally observed in 2002: T3/01, T3/03 and T3/04. When we include partial T3 sequences, we find that the alleles T3/02 and T3/05 from our initial study have not been observed in any other isolate. The expanded sample produces eight alleles seen in multiple T3 isolates, designated T3/06 to T3/13, as shown in Table 10.

There are four T3 isolates that are represented in strains maintained by the culture centers. *A. polyphaga* Panola Mountain (ATCC 30487), carries allele T3/01. *A. griffini* TIO:H37 (ATCC 50702) carries allele T3/04. *A. griffin* S-7 (ATCC 30731) carries allele T3/11. Finally, the isolate representing the type strain for *A. pearcei,* strain 205-1, exists as two ATCC cultures, ATCC 50435 and ATCC 50436, the latter representing *A. pearcei* 205-1-AX, the axenic clone derived from ATCC 50435. These cultures both carry allele T3/06, thus resulting in the allele being represented twice in the databases, while technically representing a unique T3 allele.

Among the 316 T3 isolates, 45 isolates have sequences that do not overlap the allele region, while 3 isolates had ambiguous base reads and are not included in our count of alleles. Alleles that occur more than once in the databases occur in 232 isolates. The frequencies of alleles that were in the original study or that were seen multiple times in the databases are presented in Table 10.

Only four alleles occur more than twice in the databases. Three of the original alleles make up 84% of the isolates that carry alleles found more than once, and only a single other allele has been observed more than two times. Unique, singleton alleles are carried by 36 of the 268 isolates with sequences that overlap the allele region.

### 5.2. Alleles in Acanthamoeba Sequence Type T11

Sequence type T11 is usually considered to be closely related to T3. It is represented by 152 isolates in the DNA databases—32 of which are almost complete sequences. As far as we can ascertain, no one has previously classified alleles for T11 isolates. The sequences identified with the T11 alleles that we have defined in our website are given in the Supplemental Table S4. Twelve alleles within the ASA.S1 allele region are found in multiple isolates within sequence type T11 as shown in Table 11. Two additional singleton alleles were also defined because they occur in almost complete sequences. Alleles found in multiple T11 isolates have a size range from 61 to 70 nucleotides within the allele region.

Three T11 isolates are derived from the culture collections. These include *A. hatchetti* BH-2 (ATCC 30730), which carries allele T11/01; *A. hatchetti* 4RE (ATCC PRA-115), which carries allele T11/02; *A. stevensoni* RB-F-1 (ATCC 50388), which carries allele T11/05.

Ambiguous bases were scored within the allele region of 13 isolates, while 28 isolates had sequences that did not overlap the allele region. Alleles found in multiple isolates occur in 86 of the 152 T11 isolates in the database. Five alleles are carried by more than 10 isolates, and T11 is not dominated by any single allele type, contrary to the pattern seen in the case of T3 isolates. Unique, singleton sequences were observed in 25 T11 isolates.

Although sequence types T11 and T3 are usually considered to be closely related to one another, no allele is shared between the two types. Nor are any alleles from either T3 or T11 shared with any T4 isolate.

### 5.3. Alleles in Acanthamoeba Sequence Type T5

**Figure 6:**
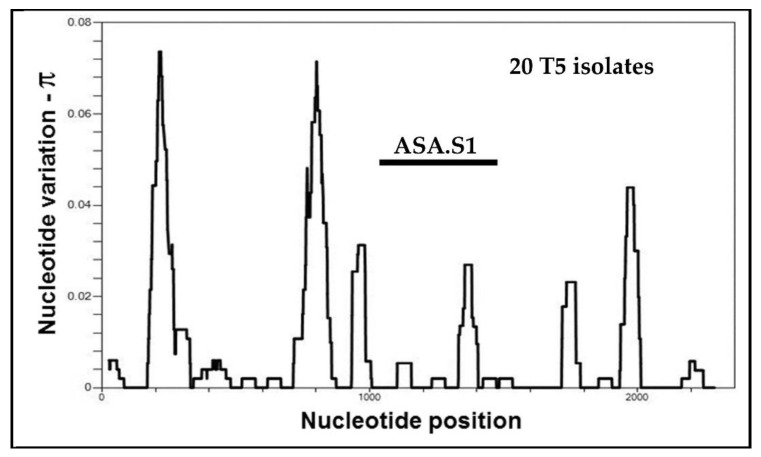
Values of nucleotide diversity (π) in a sliding window of 25 nucleotides across almost complete *Rns* sequences from 20 *Acanthamoeba* T5 isolates.

Sequence type T5 is the second most abundant sequences type after T4, with *Rns* sequences of more than 350 isolates deposited in the databases. It is the only sequence type associated with the species *A. lenticulata*. When we first examined variation of *Rns* sequences among T5 isolates for our website, we observed that variation in the ASA.S1 segment of the 18S rRNA gene was considerably lower than that observed among isolates of T3 or T4. This can be seen in Figure 6, especially when compared to Figure 2.

Consequently, we had previously expanded the regions used to categorize alleles for isolates of T5. The area that was added turned out to be the same region, the V4 hypervariable region, identified previously as a potential alternative target for identifying alleles [80], and seen as the tall peak to the left of the ASA.S1 region in Figure 6. However, as mentioned earlier, the largest numbers of isolates represent those that overlap the ASA.S1 region. Including the V4 region would remove approximately one-third of the T5 isolates that have been deposited because they do not include this second region. Therefore, to maintain comparability in this review with other sequence types, we are restricting our definition of alleles to the ASA.S1 region only.

For sequence type T5, 304 isolates can be typed for alleles in the ASA.S1 region. Fifteen alleles have been defined. The sequences are listed in Supplemental Table S5. The frequencies of alleles among T5 isolates are shown in Table 12 and follow a similar pattern to the distribution of alleles seen in T3. Three alleles (T5/01, T5/02 and T5/03) occur with moderate frequency, each exceeding 50 isolates, while only 2 other alleles (T5/06 and T5/14) occur 10 or more times. Nine of the remaining alleles occur in three or less isolates. The alleles ranged in size from 28 to 32 nucleotides, considerably shorter than alleles from the equivalent region in other sequence types. This smaller size may be an explanation for the reduced variability within the ASA.S1 region seen in Figure 6.

Twenty seven T5 sequences are among those represented by almost complete *Rns* sequences. For these isolates, 10 carry allele T5/01, including several that have been deposited in the culture centers as isolates of *A. lenticulata*, including strain SAWS87/1 (ATCC 50685), SAWS87/2 (ATCC 50686), and SAWS87/3 (ATCC 50687)—each of which are designated incorrectly in ATCC as *A*. *mauritaniensis*—and strains Jc-1 (ATCC 50428), 45 (ATCC 50703), 72/2 (ATCC 50704), 7327 (ATCC 50704). There were thirteen isolates carrying allele T5/02, including strains PD2S [AC-006] (ATCC 30841), 68-2 (ATCC 50427), NJSP-3-2 (ATCC 50429), E18-2 (ATCC 50690), 53-2 (ATCC 50691), 407-3a (ATCC 50692), 118 (ATCC 50706), and 25/1 (ATCC 50706). Single isolates with almost complete sequences carry four other of the defined alleles: T5/04, carried by isolate *A.* sp. 10: CHR-3-2-MX (ATCC 50665); T5/05, occurring in *A.* sp. 10:4C-1-MX (ATCC 50664); and alleles T5/09 and T5/13—neither of which are carried by an isolate deposited in a culture center.

In addition to those isolates that carry defined alleles, 15 isolates carried unique singleton sequences in the region of the allele, while 35 isolates did not overlap the allele region, 6 sequences contained ambiguous bases, and 2 isolates appeared to represent mixed samples.

### 5.4. Alleles in Acanthamoeba Sequence Type T15

The species *A. jacobsi* has become synonymous with isolates placed into sequence type T15. More than 150 T15 isolates have been deposited in the DNA databases. Eleven multi-isolate alleles have been identified in 125 T15 isolates. Sequences of the alleles are given in Supplemental Table S6. The frequencies of the alleles are shown in Table 13, with a single allele, T15/01, representing almost 60% of T15 isolates with defined alleles, while only one other allele currently exceeds ten isolates in the databases. Almost complete *Rns* sequences have been obtained for eight isolates, with six isolates carrying T15/01, and single isolates carrying T15/02 and T15/07. Among these isolates, the isolate that was found to carry a self-splicing intron, *A. jacobsi* isolate Pool-4-37 (Acc # KY513796), carried alleles T15/02. Three of the alleles in T15 were longer than any of the alleles seen in other sequence types, 86-87 nucleotides in length, and included the T15/02 allele associated with the intron-containing isolate. The remaining eight T15 alleles ranged from 62 to 64 bases in size.

In addition to the alleles in Table 11, 14 isolates carried unique singleton alleles, 12 isolates had sequences that did not overlap the allele region and two isolates appeared to represent mixed samples.

For *A. jacobsi*, only a single isolate has been deposited in a culture center, *A. jacobsi* 31-B [AC-005] (ATCC 30732), which carries the predominant T15 allele, T15/01.

### 5.5. Alleles in Acanthamoeba Sequence Supergroup T2/6

Sequence types T2 and T6 are the most closely related pair of sequence types within *Acanthamoeba*. We have previously argued that together the two sequence types should be considered a supergroup. This is because many isolates fall intermediate between the two types to form a total of five subtypes: T2, T2/6A, T2/6B, T2/6C and T6 [59,89]. Almost 340 isolates in the DNA databases are assigned to one of the subtypes.

The presence of alleles shared by multiple isolates was examined within the supergroup, including an analysis of how alleles are distributed into each of the five subtypes, and whether any alleles are shared between two or more of the subtypes. For context, the number of isolates whose sequences overlap the allelic region is provided in Table 14. The size of alleles in the supergroup was from 42 to 50 (as given in Supplemental Table S7).

In Table 14, it is clear that isolates that fall into the two original sequence types, T2 and T6, represent two-thirds of the isolates of the supergroup. No allele is shared between any of the subtypes, replicating the pattern seen within sequence type T4. The number of alleles found in the subtypes of the T2/6 supergroup is very similar to the number found in T4 subtypes that have equivalent numbers of isolates sampled. The observation that no allele is shared between subtypes supports a hypothesis that all five subtypes represent independent evolutionary units.

Thirty-eight isolates placed into the T2/6 supergroup are represented by almost complete sequences (Table 14). Allele diversity is reasonably well represented in the isolates with almost complete sequences in the three intermediate subtypes (seven of ten alleles represented), but less well in T2. Noteworthy, however, is the fact that none of the T6 isolates with almost complete sequences carry an allele shared with another isolate within the subtype, or within the supergroup.

The frequency of the shared alleles within each of the five subgroups is shown in Table 15. There is a distinct “most frequent allele” within T2 and T6, but alleles within the three intermediate subtypes are more evenly distributed. The allele sequences for all of the T2/6 subtypes are provided in Supplemental Table S7.

In addition to the isolates summarized in Table 14 and Table 15, four isolates contained sequences with ambiguous base reads, and 154 isolates did not have an *Rns* sequence that overlapped the allele region. There were also 43 total isolates carrying unique alleles, distributed as follows: T2 (5), T6 (21), T2/6A (3), T2/6B (9) and T2/6C (5).

A number of isolates within the T2/6 supergroup are maintained within culture collections. Among isolates in T2, two carry allele T2/01, *A. palestinensis* Reich [AC-014] (ATCC 30870), and *A*. sp. EI5 (ATCC PRA-223), while *A. pustulosa* GE3a (ATCC 50252) carries allele T2/02. Among T6 isolates, four ATCC isolates contained singleton alleles: *Comandonia operculata* CDC-149 (ATCC 50243); *A. hatchetti* 11DS (ATCC PRA-112); *A. palestinensis* 2802 (ATCC 50708); and *A.* sp. 16, RB-89-3-MX (ATCC 50669). For subtype T2/6A, one isolate from culture collections has been studied, *A*. *polyphaga* Page 45 (ATCC 30872; CCAP 1501/3b), carrying allele T2-6A/01. No isolate from subgroup T2/6B has been deposited in a culture collection. Finally, for subgroup T2/6C, *A. polyphaga* OX-1 (CCAP 1501/3c) carries allele T2-6C/01, while *A*. sp. EI4 (ATCC PRA-224) contains a unique allele.

## 6. What Do Alleles Mean for Our Understanding of the Biology of *Acanthamoeba*?

Analysis of the DNA sequence of the gene coding for the ribosomal small subunit rRNA, the 18S rRNA gene, has provided a unifying basis for the study of genetic diversity in *Acanthamoeba*. The concept of sequence types allowed a greater understanding of the diversity of the genus. Drilling down to examine the genetic diversity that exists within the gene appears to provide an additional level to our understanding of how *Acanthamoeba* is evolving.

Are alleles related to species? The answer is “probably, indirectly.” Indirectly, because single alleles are not likely to represent species; otherwise, we would be defining more than 150 species of *Acanthamoeba*. Nevertheless, groups of alleles may help to define the limits of various independent evolutionary units within the genus that do correspond to our usual concept of species. However, some concerns are raised when using alleles as a factor in our quest towards the definition of species in *Acanthamoeba.*

What are the possible problems inherent in defining alleles within subtypes? We presented data indicating that alleles are not shared between the subtypes of T4 (Figure 5) or between the subtypes of the T2/6 supergroup (Table 14). Our conclusions are based on our classification of particular isolates to a specific subgroup. There is the possibility of circular reasoning, especially when the ASA.S1 region makes up a significant proportion of the variation defining the placement of an isolate, especially for isolates with only partial sequences. We have some confidence in our classification of isolates to subtypes, based on an analysis of those isolates represented by almost complete *Rns* sequences. This is especially the case for isolates of subtypes of T4. For T4, in April 2020, our database included 404 T4 sequences that were 2000 bases in length or longer, our definition of almost complete sequences. When these sequences are classified by T4 subtype, and alleles are examined, the pattern seen in Figure 5, no allele shared between subtypes, still holds. The same is true for isolates within the T2/6 supergroup, although the number of isolates with almost complete sequences, 38, is much smaller than for T4.

Another question about the interpretation of alleles concerns those sequences seen only a single time in the DNA databases. What do such unique alleles correspond to? There are several possibilities. The most mundane explanation for many is that they simply represent DNA sequencing errors or artifacts. For instance, there are a substantial number of cases of unique alleles involving a single base insertion or deletion separating a unique allele from one of the defined alleles. Other unique alleles involve the occurrence of multiple in/del’s over the length of the allele sequence. These may be real insertions or deletions, but they also may reflect artifacts of sequencing.

Another possibility is that some unique alleles represent sequences that involve simple polymorphic sites, especially sites involving transition type differences at a site. We have observed in our own work sequences that show two peaks with slightly different heights at a site within an allele region where the site was scored for the slightly greater peak. Investigation into a few of these indicated that the site was polymorphic within the DNA mixture from the sample. We are aware of a general consensus that in studies of SNP sites in next-gen whole-genome analysis data a site in which the minor form represented less than 20% of a mixture would not be considered polymorphic. For studies of the 18S rRNA gene, since the gene occurs in an array, this rule would not be applicable. Is the sample a mixture of different isolates, or a polymorphism within a single isolate? In most cases, we opted not to pursue the issue because it did not impinge on our overall findings in a particular study. In the few cases where we did pursue an answer, we found that evidence favored polymorphism in a single isolate, rather than a mixture. If the polymorphism involves different levels of the two variants within the repetitive rRNA arrays in individual amoebae of a culture, one form may be overwhelmingly scored during sequencing, while the other form might occur rarely, or only once in the database. When the sample involved a T4 sequence type, and both forms are able to be read in the sequence and could be assigned to a known allele, we found that the alleles both fell into the same T4 subtype. This does not preclude the possibility of mixed samples from different subtypes, or even from different sequence types. However, our knowledge of the data suggests that such “unrecognized contamination” represents a very small percentage of the sequences in the DNA databases.

The final explanation for a unique allele is that it truly is a unique allele (at least in the universe of sequences deposited in the DNA databases). Population genetic theory suggests that when the allele frequency distribution is examined, the frequency of very rare alleles is directly related to the size of the population [91]. Theory also suggests that the most frequent class is most likely to be very rare alleles. Analysis of the allele frequency spectrum can be used, under certain assumptions, to gain insights into the history of a population, especially into population size changes [92,93,94], or to detect whether selection on a gene has been or is occurring [95,96,97,98]. In general, the shape of the allele frequency spectrum for each of sequence types discussed in this review show the class of unique alleles as the most common frequency class. It is possible that this occurs because most unique variants are subject to small levels of deleterious selection which prevents them from increasing in frequency. Whether this is true would require much greater study.

The identification of alleles within a sequence type or subtype allows a better tool with which to study geographic and ecological variability. Are alleles shared over the geographic range of a sequence type, or is there geographic heterogeneity that indicates that movement may be somewhat constrained by physical or climatic factors. Are some alleles more likely to be found in isolates from different ecological niches, such as soil, biofilm in water distribution systems or in fresh, brackish or seawater? Having knowledge about the occurrence of alleles will help to approach these questions. We hope to address some of these issues in a future paper.

## 7. What Do Alleles Mean for Our Understanding of Species and Speciation in *Acanthamoeba*?

What does the classification “species” mean in *Acanthamoeba*? *Acanthamoeba* is not unique. Problems in species classification abound throughout the research on single celled eukaryotes [99,100]. The Biological Species Concept (BSC) was first proposed by Ernst Mayr [101], and is usually couched in terms of reproductive separation between populations. However, the concept itself evolved over time, and additional species concepts have been proposed [102]. Microbial organisms have been especially problematic, because of questions concerning the level of possible genetic exchange between populations, or even individuals. How sexual is *Acanthamoeba*? Does (or can) genetic exchange occur by any mechanism approaching Mendelian genetic mechanisms? How important is horizontal transfer of genes to the integrity of the *Acanthamoeba* genome? Do all “taxa” of *Acanthamoeba* have the same ploidy level? What is the ploidy level of a representative member of *Acanthamoeba*? We leave the answers of these questions to others, but our impression of the literature suggests that they remain largely unanswered, although not uninvestigated.

Given the problems that might occur if we were to attempt to apply the BSC to *Acanthamoeba*, do we have other options? The most promising approach was introduced in 1980 by Niles Eldredge and Joel Cracraft [103], usually referred to as the Phylogenetic Species Concept (PSC). The Phylogenetic Species Concept considers the evolutionary relationships among organisms and relies on common ancestry and shared evolutionary history to define species. Alleles would tell us about shared evolutionary history. We could apply the principles of the PSC to deal with species classification in *Acanthamoeba*, but would need studies on additional genes to be confident of our conclusions.

A large proportion of researchers who work on *Acanthamoeba* do so because of the possibility that the amoebae affect human health. What use is it to an ophthalmologist to know a species name for the organism affecting the eye of their patient? Species designation is only of real use if we are able to show that different “species” have different potential for treatment, or affect the likelihood of different clinical outcomes. How about the utility of species names to a manager of water resources? Again, species designations would only be of use if some “species” are known to be benign, and therefore not an indication that additional water treatment, possibly expensive or difficult to employ, is required.

There certainly are reasons why names of species can be useful. Having named species that correspond with biological units can guide future research. Are all “species” equally likely to carry endosymbionts? Do they occupy different ecological niches? Can identifying members of a species allow for more carefully targeted research comparisons? We currently do not have the answers.

## 8. What Additional Approaches Are Available?

Sequence types and alleles have given us substantial insight into *Acanthamoeba*. They have not solved the problem of species. We strenuously advocate against naming new species based primarily on the sequence of the nuclear small subunit ribosomal RNA gene, even if that involves a complete or almost complete sequence. Despite the advances that we have made in the molecular study of *Acanthamoeba*, we recommend that multiple gene comparisons must be utilized.

Several additional genes provide the possibility for future multi-locus sequence typing (MLST). MLST was originally proposed for the study of bacteria [104,105]. Similar approaches have been applied to study pathogenic unicellular eukaryotes such as such as the protozoan genera *Leishmania*, *Cryptosporidium*, *Giardia*, *Trypanosoma*, and *Entamoeba*, as well as fungal genera such as *Pneumocystis*, *Candida*, and *Aspergillus* [106].

For *Acanthamoeba*, such approaches require the sequence information from additional loci. Presently, considerable progress has been made in accumulating information about the mitochondrial ribosomal small subunit (16S-like or 12S) rRNA gene [107], with 199 sequences currently available in GenBank and in our own database—most of which are from isolates that have also been studied for the nuclear 18S rRNA gene. Information also exists in the DNA databases on the mitochondrial cytochrome C oxidase subunit I (COI) gene, and the NADH dehydrogenase subunit 5 (ND5) gene. Papers reviewing these results should soon appear from our lab and from the *Acanthamoeba* lab of the Medical University of Vienna. Aspects of the COI sequences have already been used in one analysis [108].

Additional nuclear genes should also be pursued, especially given the availability of several whole-genome survey (WGS) projects on different isolates of *Acanthamoeba*. More extensive MLST analysis has only recently been attempted for *Acanthamoeba*, using a combination of 18S rRNA gene sequences and sequences from five housekeeping genes [109,110]. Utilizing data from WGS projects can expand such studies, although the number of genomes studied is still small. Such an approach has resulted in the identification of the gene for alanine-tRNA ligase, which was used to study 33 isolates, including data from the WGS of 20 isolates [111]. However, the use of the current information on WGS sequences from *Acanthamoeba* must be pursued with caution. Our studies of the nuclear 18S rRNA and mitochondrial 16S-like rRNA genes obtained from a number of WGS studies indicate that mislabeling of isolates used in some WGS studies has occurred. We mentioned above the WGS project of “*A. royreba*”—analysis of which suggests the existence of the new sequence type T22. Of the 14 WGS projects deposited in 2015 from the Centre of Genomic Research, University of Liverpool, at least 7 appear to be mislabeled as to ATCC source.

Even by expanding to whole-genome analysis, however, we may not simplify our ability to designate which of our isolates represent particular “species.” The ability to obtain complete genomic information brings new potential problems. Genome analysis offers the potential of obtaining unparalleled resolution of structure across taxonomic boundaries in species complexes. However, such resolution may have a cost. Genomic information has the potential to oversplit species if not interpreted conservatively. The potential to oversplit blurs the line between populations and species. It can complicate our ability to make simple choices of “species” vs. “not species.”

Several additional alternative approaches for the rapid identification of species have also been advocated. The most important is the concept of DNA barcoding. DNA barcoding uses short DNA sequences to taxonomically identify a specimen [112]. One of the global aims of DNA barcoding is to provide an efficient method for species-level identifications [112,113,114]. Key to the barcoding concept is the standardization of the segment of DNA used and the construction of a database of this sequence from as many taxonomically identified species as possible. For organisms such as the free-living amoebae represented by *Acanthamoeba*, work on building the library of barcodes has come slowly, and the connection between barcode and species is more tenuous. Analysis of single-celled eukaryotes has lagged that of multicellular organisms, but recent efforts have been made to encourage more work on organisms such as fungi, algae or protists [115]. There is reason to be encouraged that DNA barcoding can be useful for studies of *Acanthamoeba*, especially with the recent increase in the number of mitochondrial COI and ND5 sequences in the DNA databases. However, barcoding is primarily a method for the rapid identification of new samples and the appropriate use of DNA barcoding relies on accurate species identification. As we have documented above, species identification remains problematic in *Acanthamoeba*.

A second recent alternative method for the rapid identification of isolates is MALDI-TOF MS, a mass spectrometry technology used for rapid, automated and, hopefully, accurate microbial phenotypic identification. This technology has been used in two small studies to correctly assign *Acanthamoeba* strains into appropriate sequence types [116,117]. Whether the use of MALDI-TOF MS expands in the analysis of *Acanthamoeba* remains to be seen.

## 9. What Do We Do Next?

The standardization of species identification in *Acanthamoeba* remains an elusive goal. We suggest that the best approach will involve the FLA community moving forward on these issues. We have documented above how uncoordinated proliferation of sequence typing and allele numbering led to confusion. As our knowledge increases, we hope that an unwise proliferation of species names will not muddy future advances. There are a considerable number of isolates that have been deposited in the culture collections of ATCC or CCAP for which no molecular information has been obtained. We hope that collaboration can occur among the researchers with interest in *Acanthamoeba* that will remedy this deficiency. Perhaps the most appropriate method of coordinating advances would involve a committee composed of researchers with an interest in a rational approach to reconsidering species names for *Acanthamoeba*. Only then can we proceed with the business of cleaning up the question of what a “species” means in *Acanthamoeba*. The FLAM meetings, and the collegial group of researchers who have attended these meetings, provide a framework for us to move forward in such a process. We look forward to such an undertaking.

## Figures and Tables

**Table 1:** Species of *Acanthamoeba* described prior to 1977.

Year	Species	Type Strain	Reference
1913	*Acanthamoeba polyphaga*	CCAP 1501/3A and ATCC 30871 (*)	[18]
1930	*Acanthamoeba castellanii*	CCAP 1501/10 and ATCC 30011	[5]
1933	*Acanthamoeba palestinensis*	1547/1 and ATCC 30870	[19]
1952	*Acanthamoeba rhysodes*	CCAP 1534/3 and ATCC 30869	[7]
1954	*Acanthamoeba astronyxis*	CCAP 1534/1	[20]
1964	*Acanthamoeba comandoni*	CCAP 1501/5 and ATCC 30135	[21]
1964	*Acanthamoeba terricola*	ATCC 30134	[22]
1964	*Acanthamoeba gigantea*	None now existing	[23]
1970	*Acanthamoeba culbertsoni*	ATCC 30171	[16]
1971	*Acanthamoeba griffini*	ATCC 30731	[24]
1972	*Acanthamoeba echinulata*	ATCC 50239	[25]
1976	*Acanthamoeba lenticulata*	ATCC 30841	[26]
1977	*Acanthamoeba royreba*	ATCC 30884	[27]

(*) Original Puschkarew isolate no longer available; listed isolate is earliest deposited.

**Table 2:** New species of *Acanthamoeba* described by Pussard and Pons in 1977.

Species	Type Strain	Group
*Acanthamoeba divionensis*	ATCC 50238	II
*Acanthamoeba echinulata*	ATCC 50239 (*)	I
*Acanthamoeba lugdunensis*	ATCC 50240	II
*Acanthamoeba mauritaniensis*	ATCC 50253	II
*Acanthamoeba paradivionensis*	ATCC 50251	II
*Acanthamoeba pustulosa*	ATCC 50252	III
*Acanthamoeba quina*	ATCC 50241	II
*Acanthamoeba triangularis*	ATCC 50254	II

(*) ATCC lists strain as 278; Pussard and Pons list strain as 378.

**Table 3:** Species of *Acanthamoeba* described in the 1990s.

Year	Species	Type Strain	Group	Reference
1992	*Acanthamoeba healyi*	CDC: 1283:V013	III	[41]
1992	*Acanthamoeba jacobsi*	ATCC 30732	III	[42]
1993	*Acanthamoeba stevensoni*	ATCC 50388	II	[43]
1995	*Acanthamoeba pearcei*	ATCC 50435	II	[44]

**Table 4:** Species of *Acanthamoeba* described post-2000.

Year	Species	Type Strain	Group	Reference
2003	*Acanthamoeba sohi*	*Acanthamoeba* YM-4 (*)	II	[45]
2013	*Acanthamoeba byersi*	ATCC PRA-411	I	[46]
2015	*Acanthamoeba micheli*	BRO2-T19 (**)	II	[47]
2016	*Acanthamoeba pyriformis*	CCAP 1501/19	II or III	[48]

(*) Trophozoites deposited in the Dept. Parasitology, Yonsei University, College of Medicine; (**) Type material deposited in Medical University of Vienna.

**Table 5:** Number of isolates with one of the first 38 defined *Acanthamoeba* T4 alleles.

Allele Types	Total Observed	Allele Types	Total Observed	Allele Types	Total Observed	Allele Types	Total Observed
T4/01	59	T4/11	2	AKT4/22	244	MT4/22	7
T4/02	115	T4/12	43	AKT4/23	34	MT4/23	15
T4/03	11	T4/13	118			MT4/24	0
T4/04	43	T4/14	36	ZT4/22	25	MT4/25	29
T4/05	1	T4/15	1	ZT4/23	2		
T4/06	155	T4/16	79	ZT4/24	71	DT4/29	1
T4/07	23	T4/17	15	ZT4/25	43		
T4/08	123	T4/18	8	ZT4/26	1	RT4/31	72
T4/09	50	T4/19	2	ZT4/27	1	RT4/32	1
T4/10	83	T4/20	66	ZT4/28	1	RT4/33	48
		T4/21	78			RT4/34	9

**Table 6:** Number of isolates associated with post-2015 defined *Acanthamoeba* T4 alleles.

Allele Types	Total Observed	Allele Types	Total Observed	Allele Types	Total Observed	Allele Types	Total Observed
OT4/39	182	OT4/69	5	OT4/99	2	OT4/129	3
OT4/40	59	OT4/70	2	OT4/100	2	OT4/130	4
OT4/41	21	OT4/71	6	OT4/101	2	OT4/131	2
OT4/42	3	OT4/72	22	OT4/102	2	OT4/132	2
OT4/43	16	OT4/73	36	OT4/103	22	OT4/133	2
OT4/44	4	OT4/74	27	OT4/104	2	OT4/134	3
OT4/45	6	OT4/75	11	OT4/105	5	OT4/135	4
OT4/46	8	OT4/76	6	OT4/106	3	OT4/136	2
OT4/47	23	OT4/77	23	OT4/107	6	OT4/137	2
OT4/48	113	OT4/78	5	OT4/108	5	OT4/138	4
OT4/49	9	OT4/79	4	OT4/109	5	OT4/139	6
OT4/50	37	OT4/80	5	OT4/110	2	OT4/140	2
OT4/51	25	OT4/81	2	OT4/111	2	OT4/141	2
OT4/52	8	OT4/82	3	OT4/112	2	OT4/142	2
OT4/53	13	OT4/83	5	OT4/113	6	OT4/143	2
OT4/54	37	OT4/84	5	OT4/114	2	OT4/144	2
OT4/55	4	OT4/85	2	OT4/115	2	OT4/145	3
OT4/56	86	OT4/86	11	OT4/116	5	OT4/146	2
OT4/57	2	OT4/87	6	OT4/117	2	OT4/147	2
OT4/58	12	OT4/88	9	OT4/118	2	OT4/148	3
OT4/59	3	OT4/89	8	OT4/119	2	OT4/149	2
OT4/60	2	OT4/90	2	OT4/120	6	OT4/150	2
OT4/61	2	OT4/91	2	OT4/121	3	OT4/151	2
OT4/62	36	OT4/92	2	OT4/122	2	OT4/152	9
OT4/63	8	OT4/93	4	OT4/123	7	OT4/153	2
OT4/64	6	OT4/94	11	OT4/124	4	OT4/154	2
OT4/65	11	OT4/95	2	OT4/125	3	OT4/155	2
OT4/66	17	OT4/96	3	OT4/126	2	OT4/156	2
OT4/67	9	OT4/97	3	OT4/127	4	OT4/157	2
OT4/68	12	OT4/98	3	OT4/128	2	OT4/158	2

**Table 7:** Comparisons of DNA read from Figure 3 with two alleles identified in the DNA databases.

DNA Read	TCCTTGGCGYSKGYSKTCWWRARMMRGGSC
Allele T4/18	**TCCTTGGCGTCGGTCTTCAAAAGCCGGCGC**
Allele OT4/134	**TCCTTGGCGCGTTCGGTCTTGCAAAAGGCC**

**Table 8:** Distribution among various T4 subtypes of the number of different alleles found for “almost complete” sequences of the 18S rRNA gene from isolates of sequence type T4.

T4 Subtype	T4A	T4B	T4C	T4D	T4E	T4F	T4neff
Number of sequences	133	96	53	33	14	64	12
Number of alleles	16	9	8	10	3	7	3
Number of singletons	16	1	3	0	0	5	1

**Table 9:** Distribution among various T4 subtypes of the number of different alleles found for all sequences of the 18S rRNA gene from isolates of sequence type T4.

T4 Subtype	T4A	T4B	T4C	T4D	T4E	T4F	T4neff
sequences	1204	513	353	475	266	121	198
alleles	45	24	11	30	21	8	12
singletons	112	55	34	40	19	14	25

**Table 10:** Number of isolates associated with *Acanthamoeba* T3 alleles.

Allele Types	Total Observed	Allele Types	Total Observed	Allele Types	Total Observed
T3/01	21	T3/06	2	T3/11	2
T3/02	1	T3/07	2	T3/12	22
T3/03	101	T3/08	2	T3/13	2
T3/04	72	T3/09	2		
T3/05	1	T3/10	2		

**Table 11:** Number of isolates associated with *Acanthamoeba* T11 alleles.

Allele Types	Total Observed	Allele Types	Total Observed	Allele Types	Total Observed
T11/01	14	T11/06	11	T11/11	12
T11/02	10	T11/07	1	T11/12	4
T11/03	9	T11/08	2	T11/13	2
T11/04	13	T11/09	3	T11/14	2
T11/05	1	T11/10	2		

**Table 12:** Number of isolates associated with *Acanthamoeba* T5 alleles.

Allele Types	Total Observed	Allele Types	Total Observed	Allele Types	Total Observed
T5/01	74	T5/06	8	T5/11	2
T5/02	127	T5/07	2	T5/12	2
T5/03	51	T5/08	3	T5/13	2
T5/04	3	T5/09	2	T5/14	12
T5/05	10	T5/10	2	T5/15	3

**Table 13:** Number of isolates associated with *Acanthamoeba* T15 alleles.

Allele Types	Total Observed	Allele Types	Total Observed	Allele Types	Total Observed
T15/01	72	T15/05	2	T15/09	2
T15/02	16	T15/06	6	T15/10	9
T15/03	2	T15/07	2	T15/11	9
T15/04	3	T15/08	2		

**Table 14:** Number of isolates (total and almost complete *Rns* sequences) carrying *Acanthamoeba* T2/6 alleles.

Subtype	T2	T2/6A	T2/6B	T2/6C	T6	Total
Number of isolates	57	10	21	22	54	164
Number of alleles shared by multiple isolates	8	2	4	4	4	22
Number of “complete” sequences	17	2	7	4	8	38
Number of shared allele in “complete sequences”	4	1	4	2	0	11
Number of singletons in “complete sequences”	0	1	0	1	8	10

**Table 15:** Number of isolates associated with *Acanthamoeba* T2/6 subtype alleles.

Allele Types	Total Observed	Allele Types	Total Observed	Allele Types	Total Observed	Allele Types	Total Observed	Allele Types	Total Observed
T2/01	18	T6/01	6	T2-6A/01	3	T2-6B/01	3	T2-6C/01	8
T2/02	2	T6/02	23	T2-6A/02	4	T2-6B/02	3	T2-6C/02	3
T2/03	9	T6/03	2			T2-6B/03	2	T2-6C/03	3
T2/04	9	T6/04	2			T2-6B/04	4	T2-6C/04	3
T2/05	6								
T2/06	2								
T2/07	4								
T2/08	2								

## Supplementary Materials

The following are available online at https://www.mdpi.com/2076-0817/9/7/534/s1, Table S1: The 18S rRNS sequence from whole-genome sequence project CDEZ, sequence type T22; Table S2: Sequences for alleles occurring in multiple isolates of sequence type T4; Table S3: Sequences for alleles occurring in multiple isolates of sequence type T3; Table S4: Sequences for alleles occurring in multiple isolates of sequence type T11; Table S5: Sequences for alleles occurring in multiple isolates of sequence type T5; Table S6: Sequences for alleles occurring in multiple isolates of sequence type T15; Table S7: Sequences for alleles occurring in multiple isolates of sequence supergroup T2/6.
