# Supplementary material for: Species, Sequence Types and Alleles: Dissecting Genetic Variation in Acanthamoeba
Source: Pathogens. 2020 Jul 2;9(7):534. doi: 10.3390/pathogens9070534 (PMC7400246; doi:10.3390/pathogens9070534)
Supplement: Supplementary file 1 [file pathogens-09-00534-s001.zip › Table S1.pdf]

Table S1. 18S rRNA gene sequence for sequence type T22, Whole Genome Sequence project CDEZ, erroneously labeled "A. royreba"

```
>CDEZ Acanthamoeba royreba genome assembly
AATCGCCAAGTCCGAGAACCACGAAAAGCGCGCAGAAACGACACGCAGCGGGGGCTTGCCCGAACTTGTTTATACGGCGAGACTGC
GGATGGCTCATTAAATCAGTTATAGTTTATTTGATGGTCTCACGTCGGGCCGGCAACGGACCGACGACCCACTCTACTTGGATAAC
CGTGGTAATTCTAGAGCTAATACATGCGCAAGGTCCCGAGCGCGGGGCCGGGGCCGTTCGCGCGGTCCCGGTCCGCATGCGCGGAG
GGATGTATTTATTAGGTTAAAAACCAGCACGCACACCAAAGCATAGGCCGGTGATTCATAGTAACTCTTTCGGATCGCATCACATG
CCCCCCTCACCGGGGGCGGCGACGATTCATTCAAATTTCTGCCCTATCAACTTTCGATGGTAGGATAGAGGCCTACCATGGTCGTA
ACGGGTGACGGAGAATTAGGGTTCGATTCCGGAGAGGGAGCCTGAGAGATGGCTACCACTTCTAAGGAAGGCAGCAGGCGCGCAAA
TTACCCAATCCCGACACGGGGAGGTAGTGACGATAAATAACGATGCGGGCGCCCGACAAGGGCCTCGCAATCGGAATGAGTACGAT
TTAAACCCCTTAACGAGTAACAATTGGAGGGCAAGTCTGGTGCCAGCAGCCGCGGTAATTCCAGCTCCAATAGCGTATATTAAAGT
TGTTGCAGTTAAAAAGCTCGTAGTTGGATCTAGGGACGCGTTTCACCGAGCGGTCCGCGTCGTGCCGTTCTATGCGGTGCGGCGCG
TGGCCGTTGCGGGCTCGGTCCGCTGGAGGACCAGCGTGTCAGCCGGCCCGCCCGTCCCCTCCTTCTGGATTCCCGTTCCTGCTATT
GAGTTAGTGGGGACGTACCGCGTGCGGCGGCGGGTATCCATCCCCGTCGTGCGCGCGGAGGGGTCGTCGTCCGGCTTCGCGGCCGG
ACGGCGGCCCTCCGGGGCCCAGATCTTTTACCGTGAAAAAATTAGAGTGTTCAAAGCGGGCAGGCTTTCAATCTGCCACCGAATAC
ATTAGCATGGGATAATGGAATAGGACCCTGTCCTCCTATTTTGCGTGTTGGTTTTCGAGGACCGGGTAATGATTAATAGGGATAGT
TGGGGGCATTGATATTTAATTGTCAGAGGTGAAATTCTTGGATTTATGAAAGATCGACTTCTGCGAAAGCATCTGCCAAGGATGTT
TTCATTAATCAAGAACGAAAGTTAGGGGATCGAAGACGATCAGATACCGTCGTAGTCTTAACCATAAACGATGCCGACCAGCGATT
AGGAGACGTTGAATACAAAACACCACCGTCGGGCGGCCGTCCTTGGCTGCGTCGGGCGCGCGCGCGCCGTCCGGCGTGGCGGGGGC
GGTCCCGTCCCGGCGGCCCGGTGAATGACTCCCCTAGCAGCTTGTGAGAAATCATAAGTCTTTGGGTTCCGGGGGGAGTATGGTCG
CAAGGCTGAAACTTAAAGGAATTGACGGAAGGGCACCACCAGGAGTGGAGCCTGCGGCTTAATTTGACTCAACACGGGGAAACTTA
CCAGGTCCGGACATAGTAAGGATTGACAGATTGATAGCTCTTTCTTGATTCTATGGGTGGTGGTGCATGGCCGTTCTTAGTTGGTG
GAGCGATTTGTCTGGTTAATTCCGTTAACGAACGAGACCTTAACCTGCTAAATATGCCGCGCTAACCCGTCCATCAAAACCCACGC
GTGGCTCACGCGGTCCGCTGCAGGGCGGTGCGTCCCTTGCGAAAGGGGCGTGGCCGTCCTGCCGGCAGGGCCCGGGCGCGCGCGGG
CGGTAGGGTTCGGCGTCTGCGCTTCTTAGAGGGACTGTTGGCGTCTAGCCAGCGGAAGTTTGAGGCAATAACAGGTCTGTGATGCC
CTTAGATGTTCTGGGCCGCACGCGCGCTACACTGATTAATTCAACGAGTCCGCTTCTGTCGGGGCGCGGCGCCGCGGCGGGTCAAA
CCGTCGTGCGCGCCGCTGTTCCCCGATCGCGCCTGGGCCGATAGGTCCGGGTAATCTTTGCAAATTTAATCGTGCTGGGGATAGAT
CATTGTAATTATTGATCTTCAACGAGGAATTCCTAGTAAGCGCGAGTCATCAGCTCGCGTTGATTACGTCCCTGCCCTTTGTACAC
ACCGCCCGTCGCTCCTACCGATTGAATGGTCCGGTGAAATCCTCGGAGCCGTGGCCTCTACGCAATCCGGGCGACCGGGTTGTGAG
GTCGTCGGCGTGGCGGCAACGTCGCGTCGCATCTGGCGGCGAAGTCGATTGAACCTTACCATTTAGAGGAAGGAGAAGTCGTAACA
AGGTTTTCATCACACCACACGAGCACATCATATGGAAAGCGGGCGGGAGGAAGAAGAAGGAGAAGAGAAGCACCAAGAGGAAGAGG
ACTGCCGCCACTACTGCCGCCGCCGCCGCCGCCG
```
