# Supplementary material for: Species, Sequence Types and Alleles: Dissecting Genetic Variation in Acanthamoeba
Source: Pathogens. 2020 Jul 2;9(7):534. doi: 10.3390/pathogens9070534 (PMC7400246; doi:10.3390/pathogens9070534)
Supplement: Supplementary file 1 [file pathogens-09-00534-s001.zip › Table S2.pdf]

**Supplemental Table S2. DNA sequences for alleles in Sequence Type T4 - Sequence subtypes are listed for each allele**

| | |
|---|---|
| T4/01 - T4A | GGTGCGGTCGTCCTTGGCGTCTCGGTCCTTCACGGGGCCGGGGCGCGGGGGCGGCTTAGCCCGG |
| T4/02 - T4C | GGTGCGGTCATCCTTGGCGTTGGTCTTCAAAAGCCAGCGCGGGGGTGGCTTAGCCCGG |
| T4/03 - T4C | GGTGCGGTCGTCCTTGGCGGTTGGTCTTCAAAAGCCAGCGGCGGGGGCGGCTTAGCCCGG |
| T4/04 - T4F | GGTGCGGCTGTTCTTGGCGTCGGTTTCGGCCGGCGCGGGGATGGCTTAGCCCGG |
| T4/05 - T4C | GGTGCGGTCGTCCTTGGCGGTTGGTCTTCGAAAGCCAGCGGCGGGGGCGGCTTAGCCCGG |
| T4/06 - T4B | GGTTGCGGTCGTCCTTGGCGTCTCGGTTTCGGCCGGGGCGCGGGGATGGCTTAGCCCGG |
| T4/07 - T4A | GGTGCGGTCGTCCTTGGCGTCGGTTTCGGCCGGCGCGGGGGTGGCTTAGCCCGG |
| T4/08 - T4A | GGTGCGGTCGTCCTTGGCGTCTCGGTCCTTCACGGGGCCGGGGCGCGGGGGTGGCTTAGCCCGG |
| T4/09 - T4B | GGTGCGGTCGTCCTTGGCGTCTCGGTTTCGGCCGGGGTGCGGGGACGGCTTAGCCCGG |
| T4/10 - T4B | GGTTGCGGTCGTCCTTGGCGTCTCGGTTTCGGCCGGGGCGCGGGGACGGTTTAGCCCGG |
| T4/11 - T4A | GGCGCGGTCGTCCTTGGCCGGGTTCTCGTCCTTCACGGGGCGCGGTTCGGCGGGGGCGGCTTAGCCCGA |
| T4/12 - T4E | GGCGCGGTCGTCCTTGGCGTGTCTCGGCTTCACGGCTGGGGCGCGCGAGGGCGGTTTAGCCCGG |
| T4/13 - T4A | GGCGCGGTCGTCCTTGGCGTCTCGGTCCTTCACGGGGCCGGGGCGCGGGGGCGGCTTAGCCCGG |
| T4/14 - T4B | GGTTGCGGTCATCCTTGGCGTCTCGGTTTCGGCCGGGGCGCGGGGATGGCTTAGCCCGG |
| T4/15 - T4B | GGTTGCGGTCGTCCTTGGCGTCTCGGTTTCGGCCGGGGCGCGGGGACGGTTTACCCCGG |
| T4/16 - T4A | GGTGCGGTCGTCCTTGGCGTCGGTTTCGGCCGGCGCGGGGGCGGCTTAGCCCGG |
| T4/17 - T4C | GGTGCGGTCGTCCTTGGCGGTTGGTCTTCAAAAGCCAGCGGCGGGGGTGGCTTAGCCCGG |
| T4/18 - T4C | GGTGCGGTCGTCCTTGGCGTCGGTCTTCAAAAGCCGGCGCGGGGGTGGCTTAGCCCGG |
| T4/19 - T4C | GGTGCGGTCATCCTTGGCGTTCGGTCTTGCAAAAGGCCGGCCGCGGGGGTGGCTTAGCCCGG |
| T4/20 - T4C | GGTGCGGTCGTCCTTGGCGTTGGTCTTCAAAAGCCAGCGCGGGGGTGGCTTAGCCCGG |
| T4/21 - T4C | GGTGCGGTCGTCCTTGGCGCGTTGGTCTTCAAAAGCCAGCGCGCGGGGGCGGCTTAGCCCGG |
| AKT4/22 - T4A | GGTGCGGTCGTCCTTGGCGTCGGTCTTTCGGGGCCGGCGCGGGGACGGCTTAGCCCGG |
| AKT4/23 - T4B | GGCGCGGTCGTCCTTGGCGTCTCGGTTTCGGCCGGGGCGCGGGGACGGCTTAGCCCGG |
| MT4/22 - T4A | GGCGCGGTCGTCCTTGGCGTCTCGGTCCTTCACGGGGCCGGGGCGCGAGGGCGGCTTAGCCCGG |
| MT4/23 - T4E | GGCGCGGTCGTCCTTGGCGTTGTCGGCTTCACGGCTGGCGGCGCGAGGGCGGTTTAGCCCGG |
| MT4/25 - T4B | GGTGCGGTCGTCCTTGGCGTCTCGGTTTCGGCCGGGGCGCGGGGATGGCTTAGCCCGG |
| MT4/24 - none | GGCGCGGTCGTCCTTGGCGTGTCAGCTTCACGGCTGGCGCGCGAGGGCGGCTTTAGCCCGG |
| DT4/29 - T4E | GGCGCGGTCGTCCTTGGCGTTGTCGGCTTCACGGCTGGCGGCGCGAGGGCGGTAGAGCCCGG |
| RT4/31 - T4A | GGTGCGGTCGTCCTTGGCGTCGGTCTTTCGGGGCCGGCGCGGGGGCGGCTTAGCCCGG |
| RT4/32 - T4A | GGCGCGGTCGTCCTTGGCGTCGGTCCTTCACGGGGCCGCCGCGAGGGCGGCTTAGCCCGG |
| RT4/33 - T4D | GGCGCGGTCGTCCTTGGCGTTCGTGTTCACGCACGAGCGCGAGGGCGGCTTAGCCCGA |

| | |
|---|---|
| RT4/34 - T4E | GGCGCGGTCGTCCTTGGCGTGTCAGCTTCACGGCTGGCGCGCGAGGGCGGTTTAGCCCGG |
| ZT4/22 - T4F | GGTGCGGTCGTTCTTGGCGTCGGTTTCGGCCGGCGCGGGAGCGGCTTAGCCCGG |
| ZT4/23 - T4C | GGTGCGGTCGTCCTTGGCGTCTCGGTCCTTCACGGGGCCGGGGCGCGGGGGTGGCTTAGGCCGG |
| ZT4/24 - T4D | GGCGCGGTCGTCCTTGGCGCGTTCGTGTTCACGCACGGGCGCGCGAGGGCGGCTTAGCCCGG |
| ZT4/25 - T4D | GGCGCGGTCGTCCTTGGCGTTCGTGTTCACGCACGAGCGCGAGGGCGGTTTAGCCCGA |
| ZT4/26 - T4A | GGCGCGGTCGTCCTTGGCGTCTGTCTTTCTGGGCCGGCCCGGGGGCGGCTTAGGCTGG |
| ZT4/27 - T4A | GGTGCGGTCGTCCTTGGCGTCTCGGTCCTTCACGGGGCCGGGGCGCGGGGGTGGCTTATGCCGG |
| ZT4/28 - T4A | GGCGCGGTCGTCCTTGGCGTCGGTCTTTCTGGGCCGGCCCGGGGGCGGCTTAGCCCGG |
| OT4/39 - T4A | GGCGCGGTCGTCCTTGGCGTCGGTCCTTCACGGGGCCGGCGCGAGGGCGGCTTAGCCCGG |
| OT4/40 - T4A | GGCGCGGTCGTCCTTGGCGTCGGTCTTTCGGGGCCGGCGCGGGGGCGGCTTAGCCCGG |
| OT4/41 - T4A | GGCGCGGTCGTCCTTGGCGTCGGTCTTTCGGGGCCGGCGCGGGGACGGCTTAGCCCGG |
| OT4/42 - T4A | GGCGCGGTCGTCCTTGGCGTCGGTCTTTCGGGGCCGGCGCGGGGGCGGTTTAGCCCGG |
| OT4/43 - T4A | GGCGCGGTCGTCCTTGGCGTCGGTCCTTCGGGGCCGGCGCGAGGGCGGCTTAGCCCGG |
| OT4/44 - T4A | GGTGCGGTCATCCTTGGCGTCGGTTTCGGCCGGCGCGGGGGTGGCTTAGCCCGG |
| OT4/45 - T4B | GGTTGCGGTCGTCCTTGGCGTCTCGGTTTCGGCCGGGGCGCGGGGGCGGCTTAGCCCGG |
| OT4/46 - T4F | GGTGCGGTCGTTCTTGGCGTCGGTTTCGGCCGGCGCGGGGGCGGCTTAGCCCGG |
| OT4/47 - T4F | GGTGCGGTCGTTCTTGGCGTCGGTTTCGGCCGGCGCGGGGATGGCTTAGCCCGG |
| OT4/48 - T4Neff | GGCGCGGTCGTCCTTGGCGTCTGTCCCTTTCAACGGGGGCAGGCGCGAGGGCGGTTTAGCCCGG |
| OT4/49 - T4C | GGTGCGGTCATCCTTGGCGTTGGTCTTGCAAAAGGCCAGCGCGGGGGTGGCTTAGCCCGG |
| OT4/50 - T4D | GGCGCGGTCGCCCTTGGCGTTCGTGTTCACGCACGGGCGCGAGGGCGGCTTAGCCCGA |
| OT4/51 - T4D | GGCGCGGTCGTCCTTGGCGTTCGTGTTCACGCACGAGCGCGAGGGCGGTTTAGCCCGG |
| OT4/52 - T4D | GGCGCGGTCGTCCTTGGCGTTCGTGTTCACGCACGGGCGCGAGGGCGGTTTAGCCCGG |
| OT4/53 - T4D | GGCGCGGTCGTCCTTGGCGTTCGTGTTCACGCACGGGCGCGAGGGCGGCTTAGCCCGG |
| OT4/54 - T4D | GGCGCGGTCGTCCTTGGCGCGTTCGTGTTCACGCACGGGCGCGCGAGGGCGGTTTAGCCCGG |
| OT4/55 - T4D | GGCGCGATCGGCCTTGGCGTCTTCGTGTTCACGCACGACAGGCGCGAGGACGGTTTAGCCCGG |
| OT4/56 - T4E | GGCGCGGTCGTCCTTGGCGTGTCTCGGCTTCACGGCCGGGGCGCGCGAGGGCGGTTTAGCCCGG |
| OT4/57 - T4E | GGCGCGGTCGTCCTTGGCGTTGTCGGCTTCACGGCCGGCGGCGCGAGGGCGGTTTAGCCCGG |
| OT4/58 - T4E | GGCGCGGCCGTCCTTGGCCGTGCGTCTCGTTTCGGCGAGGCGTTTGGCGGGGGCGGCTTAGCCCGG |
| OT4/59 - T4E | GGCGCGGTCGTCCTTGGCCTGCGTCTCGTTTCGGCGAGGCGTTGGCGGGGGCGGCTTAGCCCGG |
| OT4/60 - T4E | GGCGCGGTCGTCCTTGGCCTGCGTCTCGTTTTCGGCGAGGCGTTGGCGGGGGCGGCTTAGCCCGG |
| OT4/61 - T4E | GGCGCGGTCGTCCTTGGCGCCGTCCCGTTTCGGCGGGGCGGTTGCGGGGGCGGCTTAGCCCGG |
| OT4/62 - T4E | GGCGCGGTCGTCCCTGGCTCGTCGTCTCACGACGGCGGGCGGGGGCGGTTTAGCCCGA |
| OT4/63 - T4E | GGCGCGGTCGTCCCTGGCTCGTCGTCTCACGACGGCGGGCGGGGGCGGCTTAGCCCGA |
| OT4/64 - T4D | GGCGCGGTCGTCCTTGGCGCGTTCGTGTTCACGCACGGGCGCGCGAGGGCGGTTTAGCCCCCG |

| | |
|---|---|
| OT4/65 - T4D | GGCGCGGTCGTCCTTGGCGTCTCGCGTTCGCGCGGCGCGAGGGCGGCTTACCCCGG |
| OT4/66 - T4D | GGCGCGGTCGCCCTTGGCGTTCGTGTTCACGCACGGGCGCGAGGGCGGTTTAGCCCGA |
| OT4/67 - T4D | GGCGCGGTCGCCCTTGGCGCGTTCGTGTTCACGCACGGGCGCGCGAGGGCGGTTTAGCCCGA |
| OT4/68 - T4Neff | GGCGCGGTCGTCCTTGGCGTCTCTGTCCCTTTCAACGGGGGCATGGCCGCGAGGGCGGTTTAGCCCGG |
| OT4/69 - T4A | GGCGCGGTCGTCCTTGGCGTTGGTCCTTCACGGGGCCGGCGCGAGGGCGGCTTAGCCCGG |
| OT4/70 - T4Neff | GGCGCGGTCGTCCTTGGCGGCCGTCCCTTTCAACGGGGGCGGTCGCGAGGGCGGCTTAGCCCGG |
| OT4/71 - T4D | GGCGCGGTCGTCCTTGGCGCGTTCGTGTTCACGCACGGGCGGCGCGAGGGCGGCTTAGCCCGG |
| OT4/72 - T4D | GGCGCGGTCGTCCTTGGCGCGTTCGTGTTCACGCACGAGCGCGCGAGGGCGGCTTAGCCCGA |
| OT4/73 - T4A | GGCGCGGGTCGTCCTTGGCGTCGGTCCTTCACGGGGCCGGCGCGAGGGCGGCTTAGCCCGG |
| OT4/74 - T4D | GGCGCGGTCGTCCTTGGCGTTCGTGTTCACGCACGGGCGCGAGGGCGGCTTAGCCCGA |
| OT4/75 - T4D | GGCGCGGTCGTCCTTGGCGCGTTCGTGTTCACGCACGAGCGCGCGAGGGCGGTTTAGCCCGG |
| OT4/76 - T4Neff | GGCGCGGTCGTCCTTGGCGTCTCTGTCCCTTTCAACGGGGGCAGGCGCGAGGGCGGTTTAGCCCGG |
| OT4/77 - T4Neff | GGCGCGGTCGTCCTTGGCGTCTGTGTCCCTTTCAACGGGGGCATATGGCGCGAGGGCGGTTTAGCCCGG |
| OT4/78 - T4B | GGTTGCGCGGTCATCCTTGGCGTCTCGGTTTCGGCCGGGGCGCGGGGATGGCTTAGCCCGG |
| OT4/79 - T4F | GGTGCGGCTGTTCTTGGCGTCGGTCTTTCGGCCGGCGCGGGGATGGCTTAGCCCGG |
| OT4/80 - T4E | GGCGCGGTCGTCCCTGGCAGTTTCGCTCGCGCGAGCGGGCGGGGTTGCGGGGGCGGCTTAGCCCGA |
| OT4/81 - T4A | GGTGCGGTCATCCTTGGCGTCGGTCCTTCGGGGCCGGCGCGGGGGTGGCTTAGCCCGG |
| OT4/82 - T4A | GGTGCGGTCGTCATTGGCGTCGGTCTTTAGGGGCCGGCGCGGGGACGGCTTAGCCCGG |
| OT4/83 - T4A | GGCGCGGTCGTCCTTGGCGTCGGTCTTTCGGGGCCGGCGCGAGGGCGGCTTAGCCCGG |
| OT4/84 - T4E | GGCGCGGTCGTCCTTGGCGTGTCGGCTTCACGGCCGGCGCGCGAGGGCGGTTTAGCCCGG |
| OT4/85 - T4E | GGCGCGGTCGTCCCTGGCTCGTCGTCTCACGACGGCGGGCGGGGGCGGTTAGCCCGA |
| OT4/86 - T4C | GGCGCGGTTGTCCTTGGCGGTTTGGTCTCCCTTGCAAAAGGGGGCCCAGCCCGCGGGGGCAGCTTAGCCCGG |
| OT4/87 - T4E | GGCGCGGTCGTCCCTGGCTCGTCGTTTCACGACGGCGGGCGGGGGCGGTTTAGCCCGA |
| OT4/88 - T4B | GGTTGCGCGGTCGTCCTTGGCGTCTCGGTTTCGGCCGGGGCGCGGGGATGGCTTAGCCCGG |
| OT4/89 - T4A | GGCGCGGTCGTCCTTGGCTGGTCGTCCCTCACGGGGCGGCCGGCGGGGGCGGCTTAGCCCGA |
| OT4/90 - T4D | GGCGCGGTCGTCCTTGGCGCGTTCGTGTTCACGCACGGGGGCGCGAGGGCGGTTTAGCCCGG |
| OT4/91 - T4D | GGCGCGGTCGTCCTTGGCGTTCGTGTTCACACACGAGCGCGAGGGCGGGTTACCCCGA |
| OT4/92 - T4B | GGCGCGGTCGTCCTTGGCGTCTCGGCTTCGGCCGGGGCGCGGGGACGGCTTATCCCGG |
| OT4/93 - T4D | GGCGCGGTCGTCCTTGGCGCGTTCGTGTTCACGCACGGGCGGCGCGAGGGCGGTTTAGCCCGG |
| OT4/94 - T4B | GGTGCGGTCGTCCTTGGCGTCTCGGTTTCGGCCGGGGCGCGGGGACGGCTTAGCCCGG |
| OT4/95 - T4A | GGCGCGGTCGTCCTTGGCGTCGGTCCTTCACGGGGCCGGCGCGGGGGCGGCTAGCCCTA |
| OT4/96 - T4A | GGTGCGGTCGTCCTTGGCGTCGGTCTTTCGGGGCCGGCGCGGGGGCGGCTAGCCCGG |

| | |
|---|---|
| OT4/97 - T4A | GGTGCGGTCGTCCTTGGCGTCGGTCTTTCGGGGCCGGCGCGAGGGCGGCTTAGCCCGG |
| OT4/98 - T4B | GGCGCGGTCGTCCTTGGCGTCTCGGTTTCGGCCGGGGCGCGGGACGGCTTAGCCCGG |
| OT4/99 - T4E | GGCGCGGTCGTCCCTGGCGCGTCGCCTCACGGCGGCGCGCGGGGGCGGTTTAGCCCGG |
| OT4/100 - T4E | GGCGCGGTCGTCCTTGGCGTGTCTCGGCTTCACGGCTGGGGCGCGCGAGGGCGGTAGCCCTA |
| OT4/101 - T4Neff | GGCGCGGTCGTCCTTGGCGTCTGTCCCTTTCAACGGGGGCAGGCGCGAGGGCGGTAGCCCAG |
| OT4/102 - T4Neff | GGCGCGGTCGTCCTTGGCGTCTGTGCCCCTTTCCAACGGGGGCGTGCGCGCGAGGGCGGTTCAGCCGGG |
| OT4/103 - T4B | GGCGCGGTCGTCCTTGGCGTCTCGGTTTCGGCCGGGGCGCGGGGATGGCTTAGCCCGG |
| OT4/104 - T4D | GGCGCGGTCGTCCTTGGCGCGTTCGTGTTCACGCACGGGCGCGCGAGGGCGGCTAGCCCGG |
| OT4/105 - T4A | GGTGCGGTCGTCCTTGGCGTCGGTCCTTCACGGGGCCGGCGCGGGGGACGGCTTAGCCCGG |
| OT4/106 - T4A | GGCGCGGTCGTCCTTGGCGTCTGTCCTTCACGGGGCCGGCGCGAGGGGGGTTTACCCCGG |
| OT4/107 - T4Neff | GGCGCGGTCGTCCTTGGCGTCTCTGTCCCTTTCAACGGGGGCATAGGCGCGAGGGCGGTTAGCCCGG |
| OT4/108 - T4A | GGTGCGGTCGTCCTTGGCGTCTCGGTCCTTCACGGGGCCGGGGCGCGGGGGCGGCTAGCCCGG |
| OT4/109 - T4A | GGCGCGGTCGTCCTTGGCGTCGGTCCTTCACGGGGCCGGCGCGAGGGCGGCTAGCCCTG |
| OT4/110 - T4E | GGCGCGGTCGTCCTTGGCGTGTCTCGGCTTCACGGCCGGGGCGCGCGAGGGCGGTTAGCCCTA |
| OT4/111 - T4A | GGCGCGGTCGTCCTTGGCGTCGGTCCTTCACGGGGCCGGCGCGAGGGCGGCTAGCCCGT |
| OT4/112 - T4Neff | GGCGCGGTCGTCCTTGGCGTCTGTCCCTTGGGGCAGGCGCGAGGGCGGTTTAGCCCGG |
| OT4/113 - T4E | GGCGCGGTCGTCCTTGGCGTTCGTCAGCTTCACGGCCGGCGGGCGCGAGGGCGGTTTAGCCCGG |
| OT4/114 - T4A | GGCGCGGTCGTCCCTGGCGTCGCGCAAGCGGCGCGGGGGCGGCTTAGCCCGA |
| OT4/115 - T4D | GGCGCGGTCGTCCTTGGCGTTTGTGTTCTCGCACGAGCGCGAGGGCGGGTTAGCCCGA |
| OT4/116 - T4A | GGCGCGGTCGTCCTTGGCGTCGGTTTCGGCCGGCGCGGGGGCGGCTTAGCCCGG |
| OT4/117 - T4A | GGCGCGGTCGTCCTTGGCGTCTCGGTCCTTCACGGGGCCGGGGCGCGGGGGCGGCTTAACCCGG |
| OT4/118 - T4C | GGTGCGGTCATCCTTGGCGTTGGTCTTGCAAAAGGCCAGCGCGGGGGTGGCTTATCCCGG |
| OT4/119 - T4Neff | GCTGCGGTCGTCCTTGGCGTCTGTCCCTTTCAACGGGGGCAGGCGCGAGGGCGGTTTAGCCCGG |
| OT4/120 - T4A | GGTTGCGGTCGTCCTTGGCGTCGGTCTTTCAACTGGGGGGCCGGCGCGGGGACGGCTTAGCCCGG |
| OT4/121 - T4Neff | GGCGCGGTCGTCCTTGGCGTCTGTCCCTTTCAACGGGGGCATGGCGCGAGGGCGGTTTAGCCCGG |
| OT4/122 - T4D | GGCGCGGTCGTCCTTGGCGCGTTCGTGTTCACGCACGGGGGCGCGAGGGCGGCTTACCCCGG |
| OT4/123 - T4F | GGTGCGGTCGTTCTTGGCGTCGGTTTCGGCCGGCGCGGGGACGGCTTAGCCCGG |
| OT4/124 - T4D | GGCGCGGTCGTCCTTGGCGTTCGTGTTCACGCACGGGCGCGAGGGCGGCTTACCCCGA |
| OT4/125 - T4D | GGCGCGGTCGCCCTTGGCGCGTTCGTGTTCACGCACGGGCGCGCGAGGGCGGCTTAGCCCGG |
| OT4/126 - T4D | GGCGCGGTCGTCCTTGGGGTTCGTGTTCACGCGCGAGCGCGAGGGGGGTTTAGCCCGG |
| OT4/127 - T4F | GGTGCGGCTGTTCTTGGCGTCGGTTTCGGCCGGCGCGGGGACGGCTTAGCCCGG |
| OT4/128 - T4A | GGGGCGGTCGTCCTTGGCGTCTGTTTCGGCCGGTGCGGGGGTGGCTTATCCCGG |
| OT4/129 - T4A | GGCGCGGTCGTCCTTGGCGTCTCGCGTTCGCGCGCGGCGCGAGGGCGGCTTAGCCCGG |
| OT4/130 - T4A | GGCGCGGTCGTCCTTGGCCGCGGGTTCGTCCTTCACGGGGCGGGTTCCGGCGGGGGCGGCTTAGCCCGA |

| | |
|---|---|
| OT4/131 - T4D | GGCGCGGTCGTCCTTGGCGCGTTCGTGTTCACGCACGGGCGCGCGAGGGCGGCTTAGCCCGA |
| OT4/132 - T4A | GGCGCGGTCGTCCTTGGCGTCTCGGTCCTTCACGGGGCCGGGGCGCGGGGGCGGCTTACCCCGG |
| OT4/133 - T4B | GGCGCGGTCGTCCTTGGCGTCTCGGTTTCGGCCGGGGCGCGGGGATGGCTTAACCCGG |
| OT4/134 - T4C | GGTGCGGTCATCCTTGGCGCGTTCGGTCTTGCAAAAGGCCAGCGCGCGGGGGTGGCTTAGCCCGG |
| OT4/135 - T4B | GGTTGCGGTCGTCCTTGGCGTCTCGGTTTCGGCCGGGGCGCGGGGACGGCTTAGCCCGG |
| OT4/136 - T4A | GGTGCGGTCGTCCTTGGCGTCTCGGTCCTTCACGGGGCCCGGGCACGGGGGTGGCTTATCCCGG |
| OT4/137 - T4F | GGTGCGGTCGTTCTTGGCGTCGGTCTTTCGGCCGGCGCGGGAGCGGCTTAGCCCGG |
| OT4/138 - T4B | GGTTGCGGTCGTCCTTGGCGTCTCGGTTCGGCCGGGCGCGGGACGTTAGCCCGG |
| OT4/139 - T4B | GGTTGCGGTCGTCCTTGGCGTCTCGGTTTCGGCCGGGGCGCGGGACGTTAGCCCGG |
| OT4/140 - T4B | GGTTGCGGTCGTCCTTGGCGTCTCGGTTTCGGCCGGGGCGCGGGACGGTAGCCCGG |
| OT4/141 - T4B | GGTGCGGTCGTCCTTGGCGTCTTTGTTTCGGCCGGGGTGCGGGGACGGCTTAGCCCGG |
| OT4/142 - T4D | GGCGCGGTCGTCCTTGGCGTTCGTGTTCACGCACGAGCGCGAGGGCGGCTTAGCCCGG |
| OT4/143 - T4A | GGCGCGGTCGTTCCTGGCGTCGCGCAAGCGGCGCGGGAGCGGCTTAGCCCGA |
| OT4/144 - T4A | GGTGCGGTCGTCCTTGGCGTCGGTCTTTCTGGGCCGGCGCGGGGACGGTTTAGCCCGG |
| OT4/145 - T4B | GGTTGCGGTCGTCCTTGGCGTCTCGGTTTCGGCCGGGCCGCGGGGACGCTTTAGCCCGG |
| OT4/146 - T4A | GGTGCGGTCGTCCTTGGCGTCGGTTTCGGGCCGGCGCGGGGGGCGGCTAGCCCCG |
| OT4/147 - T4A | GGTGCGGTCGTCCTTGGCGTCGGTCTTTCGGGGCCGGCGCGAGGGTGGCTTAGCCCGG |
| OT4/148 - T4E | GGCGCGGTCGTCCTTGGCGTGTCTCGGCTTCACGGCTGGGGCGCGCGAGGGCGGTTAGCCCGG |
| OT4/149 - T4A | GGTGCGGTCGTCCTTGGCGTCTCGGTCCTTCACGGGGCCGGGGCGCGGGGGCGGCTAGCCCTG |
| OT4/150 - T4A | GGCGCGGTCGTCCTTGGCGTCTCTCGGTCCTTCACGGGGCCGGGGCGCGGGGGCGGCTTAGCCCGG |
| OT4/151 - T4E | GGCGCGGTCGTCCTTGGCGTGTCTCGGCTTCACGGCTGGCGCGCGAGGGCGGTTTAGCCCGG |
| OT4/152 - T4D | GGCGCGGGTCGTCCTTGGCGCGTTCGTGTTCACGCACGGGCCGG |
| OT4/153 - T4D | GGCGCGGTCGTCCTTGGCGTCTGTGTCCCTTTCAACGGGGGCATGGCGCGAGGGCGGTTTAGCCCGG |
| OT4/154 - T4D | GGCGCGGTCGTCCTTGGCGCGTTCGTGTTCACGCACGGGCGGCGCGAGGGCGGCTTACCCCGG |
| OT4/155 - T4B | GGCGCGGTCGTCCTTGGCGTCTCGGTTTCTGGCGGGGCCCGGGGGCGGGTTAGGCTTG |
| OT4/156 - T4B | GGCGCGGTCGTCCTTGGCGTCTCGGTTTCTGGCGGGGCCCGGGGGCGGGTTAGGCCGG |
| OT4/157 - T4B | GGCGCGGTCGTCCTTGGCGTCTCGGTTTCGGGGGGGGCGCGGGGGCGGCTTAGCCCGG |
| OT4/158 - T4B | GGCGCGGTCGTCCTTGGCGTCTCGGTTTTTGGCGGGGCCGGGGGGCGGGTTAGGCCTG |
