# Supplementary material for: Species, Sequence Types and Alleles: Dissecting Genetic Variation in Acanthamoeba
Source: Pathogens. 2020 Jul 2;9(7):534. doi: 10.3390/pathogens9070534 (PMC7400246; doi:10.3390/pathogens9070534)
Supplement: Supplementary file 1 [file pathogens-09-00534-s001.zip › Table S3.pdf]

Supplemental Table S3. DNA sequences for alleles in Sequence Type T3

T3/01	GCGATTGCGGTCGTCTTTGGTGTCGCTCACAAGGCGGCATCGGGACGGCTTAGCTCGCA
T3/02	GCGATTGCGGTCGTCTTTGGTGTCGCTCACAAGGCGGCATCGGGGCGGCTTAGCTCGCA
T3/03	GCGCATTGCGGTCGTCTTTGGTGTCGCTCACAAGGCGGCACCGGGGCGGCTTAGCTCGCA
T3/04	GCGCATTGCGGTCGTCTTTGGTGTGTCGCTCACAAGGCGGCATCATCGGGACGGCTTAGCTCGCA
T3/05	GCGATTGCGGTCGTCTTTGGTGTCGTCCACAGCGATGTGGGCGGCATCGGGATGGCTTAGCTCGCA
T3/06	GCGATTGCGGTCGTCTTTGGTGCCATCCACAGCGTGTGTGGTGGCATCGGGATGGCTTAGCTCGCA
T3/07	GCGCATTGCGGTCGTCTTTGGTGTCGCTCACAAGGCGGCATCGGGACGGCTTAGCTCGCA
T3/08	GCGATTTGCGTTCTCTTTTGGGGGTGCTTAACAAGGGGGGGTCATGCGGATTGCTTCGCTTGGA
T3/09	GCGCATTGCGGTCGTCTTTGGTGTGTCGCTCACAAGGCGGCATCATCGGGACGGCTAGCTCGCA
T3/10	GCGCATTGCGGTCGTCTTTGGTGTGTCGCTCACAAGGCGGCATCATCGGGACGGCTAAGCTCGGCA
T3/11	GCGCATTGCGGTCGTCTTTGTTGTCGCTCACAAGGCGGCACCGGGGCGGCTTAGCTCGCA
T3/12	GCGCATTGCGGTCGTCTTTGGTGTCGCTCACAAGGCGGCACCGGGGCGGCTCCGTATCA
T3/13	GCGCATTGCGGTCGTCTTTGGTGTCGCTCACAAGGCGGCACCGGCGCGGCTTAGCTCGCA
