# Supplementary material for: Species, Sequence Types and Alleles: Dissecting Genetic Variation in Acanthamoeba
Source: Pathogens. 2020 Jul 2;9(7):534. doi: 10.3390/pathogens9070534 (PMC7400246; doi:10.3390/pathogens9070534)
Supplement: Supplementary file 1 [file pathogens-09-00534-s001.zip › Table S4.pdf]


Supplemental Table S4. DNA sequences for alleles in Sequence Type T11

T11/01	CGGTGCGGTCGTCCTTGGCGCGTCGTGGCTTGCTGCGGCGTGCGAGGGCGGTTTAGCCTGA
T11/02	CGGTGCGGTCGTCCTTGGCGCGTCGCGGCTTGCCGCGGCGTGCGAGGGCGGTTTAGCCTGA
T11/03	CGGTGCGGTCGTCCTTGGCGCGTCGTGGCTTGCTGCGGCGTGCGAGGGCGATTTAGCCTGA
T11/04	CGGTGCGGTCGTCCTTGGCGCGTCGCGGCTTGCCGTGGCGTGCGAGGGCGGTTTAGCCTGA
T11/05	CGGTGCGGTCGTCCTTGGCACGTCGTGGCGCAAGCCATGGCGGGCGAGGGCGATTTAGCCTGA
T11/06	TGGTGCGGTCGTCCTTGGCATTTGGTCGTGGCGCAAGTCACGGCCAGGTGTGAGGACGGTTTAGCCTGA
T11/07	TGGTGCGGTCGTTCTTGGCATTTGTCGTGGCGCGAGTCACGGCAGGTGCGGGGACGGTTTAGCCTGA
T11/08	TGGTGCGGTCGTTCCTTGGCATTTGGTCGTGGCGCAAGTCACGGCCAGGTGTGAGGACGGTTTAGCCTGA
T11/09	TGGTGCGGTCGTCCTTGGCATTTGGTCGTGGCGCGAGTCACGGCCAGGTGCGGGGACGGTTTAGCCTGA
T11/10	CGGTGCGGTCGTCCTTGGCACGTCATTGGGCGCAAGCCCGTGGCGGGCGAGGGCGGTTTAGCCTGA
T11/11	CGGTGCGGTCGTCCTTGGCATTTGTCGTGGCGCAAGTCACGGCAGGTGTGAGGATGGTTTAGCCTGA
T11/12	TGGTGCGGTCGTTCTTGGCATTTGGTCGTGGCGCGAGTCACGGCCAGGTGTGAGGACGGTTTAGCCTGA
T11/13	CGGTGCGGTCGTCCTTGGCATTTGGTCGTGGCGCAAGTCACGGCAGGTGCGAGGACGGTTTAGCCTGA
T11/14	CGGTGCGGTCGTCCTTGGCATTTGTCGTGGCGCGAGTCACGGCAGGTGCGGGGACGGTTTAGCCTGA
