# Supplementary material for: Species, Sequence Types and Alleles: Dissecting Genetic Variation in Acanthamoeba
Source: Pathogens. 2020 Jul 2;9(7):534. doi: 10.3390/pathogens9070534 (PMC7400246; doi:10.3390/pathogens9070534)
Supplement: Supplementary file 1 [file pathogens-09-00534-s001.zip › Table S5.pdf]


Supplemental Table S5. DNA sequences for alleles in Sequence Type T5

T5/01	GCCGTTAATCCTTTCGGGGGTTAATGGT
T5/02	GCTGTTAATCCTTTCGGGGGTTAATAGT
T5/03	GCTGTTAGTCCTTTCGGGGATTAATAGT
T5/04	GCCGTTAATCCTTTCGGGGATTAATGGT
T5/05	GCTGTTAATCCTTTCGGGGATTAATAGT
T5/06	GCCGTTAATCCTTTTCAACGGGGGTTAACGGT
T5/07	GCCATTGATCCTCTCGGGGGTTAATGGT
T5/08	GCCGTTAATCCTTTTTCGGGGGTTAACGGT
T5/09	GCCGTTAATCCTTTCGGGGGTTAACGGT
T5/10	GCTGTTAGTTCTCTTTCGGGGACTAATAGT
T5/11	GCATGTTAATACCTTTCGGGGGTTAATAGT
T5/12	GCACGTTAATACCTTTCGGGGGTTAATGGT
T5/13	GCTGTTAATCCCCTTTCGGGGGTTAATAGT
T5/14	GCTATTAATCCTTTCGGGGGTTAATGGT
T5/15	GCTGTTAATCCTTTTCAACGAGGGTTAATGGT
