# Supplementary material for: Species, Sequence Types and Alleles: Dissecting Genetic Variation in Acanthamoeba
Source: Pathogens. 2020 Jul 2;9(7):534. doi: 10.3390/pathogens9070534 (PMC7400246; doi:10.3390/pathogens9070534)
Supplement: Supplementary file 1 [file pathogens-09-00534-s001.zip › Table S6.pdf]

Supplemental Table S6. DNA sequences for alleles in Sequence Type T15

T15/01	GTCGGGTCGGCCGTCCTTGGCCGTCGTGCGCGGCGACGCGCGCGGCGGCGAGGGCGGTGTCTGC
T15/02	GTTGGGTCGTGGCGGGCCGTGCCCCGCGGTCCGTCGTGCGCGGCGACGCGTGCGGCGGCCACGGGGACCGCGGTCCGTATCAGCCAC
T15/03	GTTGGGTCGTGGCGGGCCGTGCCCCGCGGTCCGTCTTGCGCGGCGACACGTGCGGCGGCCACGGGGACCGCGGTCCGTATCAGCCAC
T15/04	GTCGGGTCGGCCGTCCTTGGCCGTCGTGCGCGGCGACGCGCGCGGCGGCGAGGGCGGTCTGC
T15/05	GTCGGGTCGGCCGTCCTTGGCCGTCGTGCGCGGCGACGCGTGCGGCGGCGAGGGCGGTGTCTGC
T15/06	GTTGGGTCGTGGCGGGCCGTGCCCCGCGGTCCGTCGTGCGCGGCGAGCGTGCGGCGGCCACGGGGACCGCGGTCCGTATCAGCCAC
T15/07	GTCGGGTCGGCCGTCCTTGGCCGTCGTGCGCCGCGAGGCGTGCGGCGGCGAGGGCGGCGTCTGC
T15/08	GTCGGTTCGGCCGTCCTTGGCCGTCGTGCGCGGCGACGCGCGCGGCGGCGAGGGCGGTGTCTGC
T15/09	GTTGGGTCGGCCGTCCTTGGCCGTCGTGCGCGGCGACGCGCGCGGCGGCGAGGGCGGTGTCTGC
T15/10	GTCGGGTCGGCCGTCCTTGGCCGTCGTGCGCGGCGAGGCGCGCGGCGGCGAGGGCGGCGTCTGC
T15/11	GTCGGGTCGGCCGTCCTTGGCCGTCGTGCGCCGCGACGCGCGCGGCGGCGAGGGCGGTGTCTGC
