# Supplementary material for: Species, Sequence Types and Alleles: Dissecting Genetic Variation in Acanthamoeba
Source: Pathogens. 2020 Jul 2;9(7):534. doi: 10.3390/pathogens9070534 (PMC7400246; doi:10.3390/pathogens9070534)
Supplement: Supplementary file 1 [file pathogens-09-00534-s001.zip › Table S7.pdf]


Supplemental Table S7. DNA sequences for alleles in Sequence Type T4 - Sequence subtypes are listed for each allele

---

T2/01	ACATAATTCAGTACATGGCCAGTCAAATGGTTGTGTATTGTTTGTGTA
T2/02	ATATAATTCAGTACATGGCCAGTCAAATGGTTGTGTATTGTTTGTGTA
T2/04	ACATAATTCAGTATATGGCCAGTCAAATGGTTGTATATTGTTTGTGTA
T2/03	GTATAATTCAGTATATGGCCAGTCAAATGGTTGTATATTGTTTGTATA
T2/05	GTATAATTCAGTACATGACCAGTCAAATGGTTGTGTATTGTTTGTATA
T2/06	GTATAATTCAGTATATGGCCAGTCAAATGGTTGTATATTGTTTGTGTA
T2/07	GTATAATTCAGTATATGGCCAGTCAAATGGTTGTATATTGTTTGTACA
T2/08	GCATAATTCAGTATATGGCCAGTCAAATGGTTGTATATTGTTTGTGTA
T26A/01	AGATAATTCATTATATGGCTTCACGGCTGTATAGTGTTTGTCTT
T26A/02	CGATAATTCAGTATATGGCTTCACGGATGTATATTGTTTGTCTT
T26B/01	ACATAATTCAGCATATGGCATCCTCACGGGTGTTGTGTGTTGATTGTGTA
T26B/02	GCATAATTCAATATATGGCATCCTCACGGGTGTTGTATGTTGATTGTGTA
T26B/03	GTATAATTCAATATATGGCATCCTCACGGGTGTTGTATGTTGATTGTATA
T26B/04	GTATAATTCAATATATGGCACCTTCACGGGTGTTGTATGTTGATTGTATA
T26C/01	ATATAATTCAGTATATGGTCAGCAATGGCTGTATATTGTTTGTATA
T26C/02	ATATAATTCAATATATGGCTTCACGGCTGTATGTTGTGTATA
T26C/03	ATATAATTCAGTACATGGTCAGCAATGGCTGTGTATTGTTTGTATA
T26C/04	ATATAATTCAGTATATGGTCAGCAATGGCTGTGTATTGTTTGTATA
T6/02	ATATAATTCAGTATATGGCTAGCAATGGCTGTATATTGTTTGTGTA
T6/01	ATATAATTCAGTATATGGTCAGTCAAATGGCTGTGTATTGTTTGTATA
T6/03	ATATAATTCAGTGTATGGCTTCACGGCTGTACATTGATTGTATA
T6/04	ATATAATTCAGTGTATGGCTTCACGGCTGTGCATTGATTGTATA
